# Periodontal disease and endocrine imbalance: a molecular pathways perspective

**DOI:** 10.3389/fdmed.2026.1886028

**Published:** 2026-07-17

**Authors:** Mireya Martínez-García, Enrique Hernández-Lemus

**Affiliations:** 1 Dental Public Health Department, Division of Graduate Studies and Research, School of Dentistry, Universidad Nacional Autónoma de México, Mexico City Mexico; 2 Computational Genomics Division, National Institute of Genomic Medicine, México City, México

**Keywords:** endocrine disorders, metabolic and signaling pathways, oral health, pathway crosstalk, periodontal disease, periodontal-endocrine crosstalk, systemic inflammation

## Abstract

Periodontitis is a chronic multifactorial inflammatory disease whose pathological consequences extend well beyond the oral cavity to affect systemic health through the dissemination of pro-inflammatory mediators, bacteremic episodes, and the amplification of low-grade systemic inflammation. Concurrently, the endocrine system functions as a central regulator of periodontal tissue homeostasis, with hormone receptors for estrogens, androgens, glucocorticoids, insulin, thyroid hormones, and vitamin D expressed across the principal cell populations of the periodontium, including gingival fibroblasts, PDL cells, osteoblasts, osteoclasts, and epithelial cells. This narrative review synthesizes current evidence on the molecular pathways mediating the bidirectional relationship between periodontal disease and endocrine imbalance, integrating experimental, clinical, and systems-level bioinformatics data to provide a comprehensive mechanistic framework for this interaction. We examine how estrogen deficiency, progesterone excess, androgen dysregulation, insulin resistance, adipokine imbalance, thyroid dysfunction, glucocorticoid excess or deficiency, and perturbations of the parathyroid hormone and vitamin D axes each contribute to periodontal susceptibility through distinct but convergent molecular mechanisms involving NF-κB activation, NLRP3 inflammasome-driven IL-1β and IL-18 maturation, cytokine network dysregulation, oxidative stress amplification, and disruption of the RANKL/OPG balance governing alveolar bone homeostasis. We also discuss key hub genes including TNF, IL6, LEP, NOS3, STAT3, and VEGFA as molecular nodes that simultaneously participate in endocrine and periodontal signaling, and we highlight the PI3K/Akt, AGE-RAGE, JAK-STAT, Th17 differentiation, and HIF-1 pathways as the most significantly enriched molecular routes of endocrine-periodontal crosstalk. The RANKL/OPG axis emerges as a central integrator of hormonal and inflammatory inputs to alveolar bone metabolism, with estrogen, PTH, glucocorticoids, vitamin D, leptin, and adiponectin each converging on this molecular switch through receptor-mediated mechanisms that are additive to the inflammatory RANKL upregulation driven by IL-1β, IL-6, IL-17, and TNF-α in active periodontal lesions. Emerging pharmacological agents at the endocrine-periodontal interface, including GLP-1 receptor agonists, denosumab, statins, vitamin D supplementation, and specialized pro-resolving mediators, offer mechanistically grounded opportunities for integrated therapeutic strategies that address both oral and systemic disease simultaneously. The diagnostic and therapeutic implications of these molecular relationships demand a fundamental reorientation of clinical management toward interdisciplinary models in which periodontal assessment is incorporated into the evaluation of patients with endocrine disorders, endocrine status is systematically characterized in patients with severe or treatment-refractory periodontitis, and therapeutic decisions in both specialties are made with explicit awareness of the shared molecular architecture linking them.

## 1 Introduction

Periodontitis is a chronic, multifactorial inflammatory disease that affects the supporting structures of the teeth and represents one of the most prevalent oral conditions in adults worldwide. Although historically framed as a localized oral ailment, compelling evidence accumulated over the past two decades has firmly established periodontitis as a condition with profound systemic implications [1–4]. Its pathogenesis is initiated by dysbiotic polymicrobial biofilms that provoke an uncontrolled host immune response; however, it is the dysregulated inflammatory reaction—rather than the microbial challenge alone—that drives connective tissue destruction, alveolar bone resorption, and the spillover of inflammatory mediators into the systemic circulation [5, 6]. Pro-inflammatory cytokines including interleukin (IL)-1β, IL-6, IL-17, and tumor necrosis factor-alpha (TNF-α), locally overproduced in periodontal tissues, can disseminate through ulcerated pocket epithelium and bacteremic episodes, thereby amplifying low-grade systemic inflammation and contributing to the exacerbation of distant organ pathology [7, 8].

The systemic reach of periodontal inflammation has been documented across a remarkable breadth of disease categories. Epidemiological, clinical, and molecular studies have consistently linked periodontitis with heightened risk or worsened outcomes in cardiovascular diseases, type 2 diabetes mellitus, rheumatoid arthritis, neurodegenerative conditions, adverse pregnancy outcomes, and several cancers, among others [3, 9]. These associations are not merely coincidental; they reflect shared inflammatory pathways, common genetic susceptibility loci, dysbiotic microbiome interactions, and molecular crosstalk between immune mediators that operate across organ systems [1]. A comprehensive molecular comorbidity analysis of periodontitis, employing gene–disease association mining across curated databases, revealed that the periodontitis diseasome encompasses over 80 distinct disease conditions sharing associated genes, proteins, or biological pathways with periodontitis, organized in a highly interconnected network whose core is dominated by inflammatory, immune, and metabolic signaling molecules [4].

**Figure 1:**
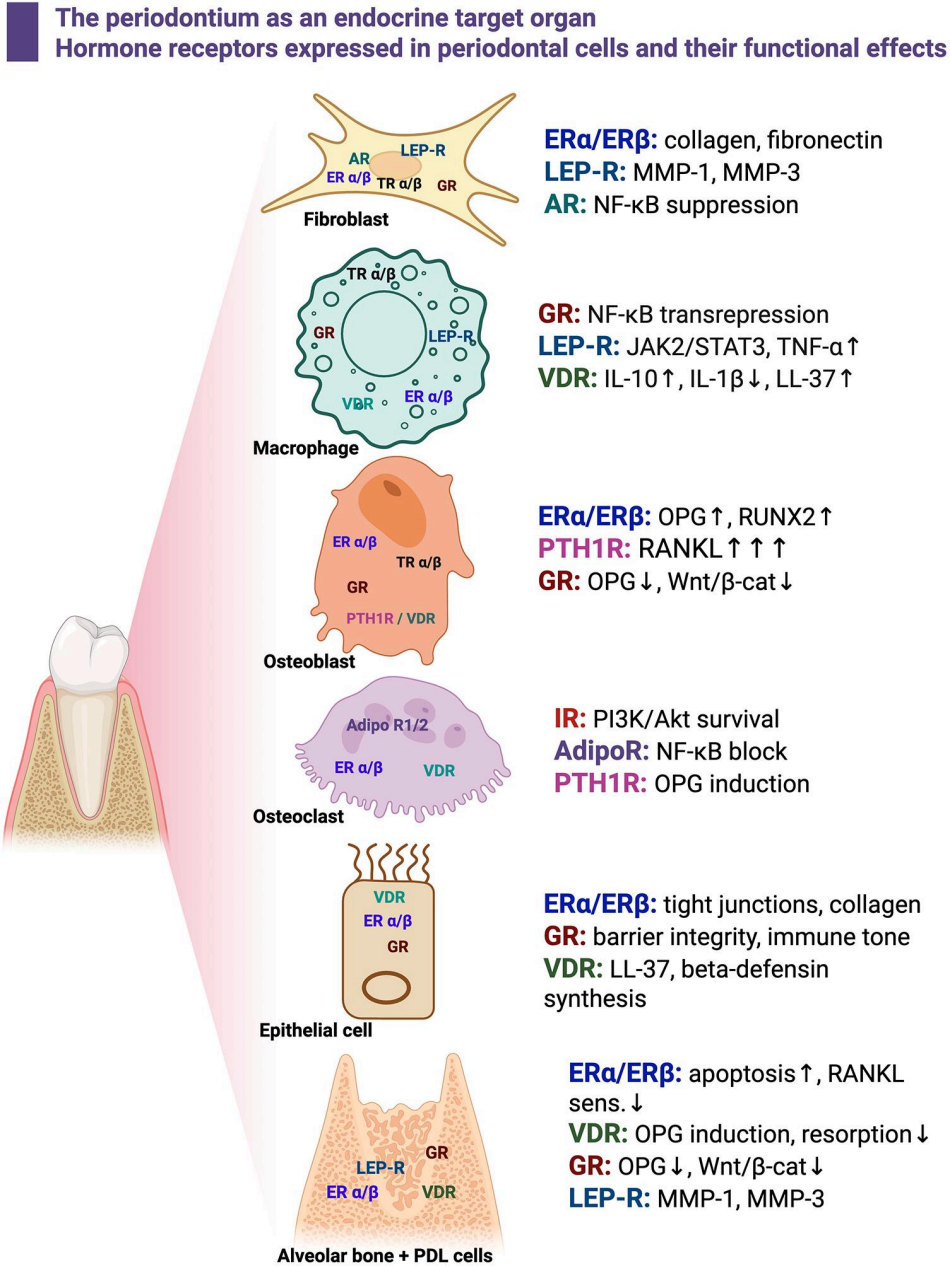
The periodontium as an endocrine target organ: hormone receptors expressed in periodontal cells and their functional effects. Created in https://BioRender.com.

Within this broad landscape of comorbidities, endocrine disorders occupy a particularly prominent position. The endocrine system functions as a central regulator of homeostatic processes throughout the organism, modulating immune cell behavior, bone remodeling dynamics, tissue repair capacity, and the production of inflammatory mediators [2, 10]. Hormones do not merely orchestrate reproductive and metabolic functions; they actively shape the inflammatory microenvironment of tissues throughout the body, including the periodontium (Figure 1). Estrogen receptors alpha and beta (ERα and ERβ), androgen receptors, glucocorticoid receptors, vitamin D receptors (VDR), thyroid hormone receptors, and insulin receptors have all been identified in periodontal fibroblasts, epithelial cells, osteoblasts, osteoclasts, and PDL cells, conferring on these tissues a direct responsiveness to hormonal fluctuations [11, 12]. Accordingly, conditions characterized by hormonal excess, deficiency, or dysregulation are expected to alter periodontal tissue homeostasis in clinically meaningful ways.

In this context, we will discuss about endocrine imbalance, broadly defined here to denote any deviation from physiological hormonal homeostasis, whether arising from pathological endocrine disease, normal physiological transitions (e.g., puberty, pregnancy, menopause), or pharmacological hormone exposure (e.g., corticosteroid therapy, hormonal contraception, hormone replacement therapy), that alters the hormonal signals reaching periodontal tissues.

The molecular underpinnings of this relationship are beginning to be elucidated. As detailed in Section 9, network-based analyses of the periodontitis diseasome reveal that several endocrine and metabolic conditions—including obesity, non-insulin-dependent diabetes mellitus, polycystic ovary syndrome, adrenal gland hypofunction, and secondary hyperparathyroidism—share significant numbers of associated genes with periodontitis, and identify hub genes such as LEP, NOS3, and STAT3 as molecular bridges between periodontal and endocrine pathology [4]. These findings situate the endocrine-periodontal relationship not as an isolated clinical observation, but as an expression of deep molecular interconnection across shared biological networks (Figure 1 shows some of the main molecular pathways altered in several cell types in the periodontium).

Hormonal signals exert their influence on periodontal tissues through multiple, often intersecting pathways—sex steroid, adipokine, insulin, thyroid, parathyroid, and glucocorticoid signaling each modulate distinct but convergent aspects of cytokine production, immune cell behavior, and the RANKL/OPG balance governing alveolar bone remodeling, as examined in detail in Sections 3 through 7. Taken together, these mechanisms converge on a shared molecular architecture of inflammation, immune dysregulation, and bone metabolism that is perturbed in both periodontal disease and endocrine dysfunction.

The NLRP3 (NOD-, LRR- and pyrin domain-containing protein 3) inflammasome has recently emerged as a central integrator of metabolic danger signals, microbial pattern recognition, and endocrine inputs that are simultaneously dysregulated in both periodontal disease and endocrine disorders [13]. NLRP3 is activated downstream of NF-κB priming by pathogen-associated molecular patterns (PAMPs) and damage-associated molecular patterns (DAMPs), including lipopolysaccharide from periodontal pathogens, reactive oxygen species (ROS), advanced glycation end products (AGEs), and saturated free fatty acids characteristic of the metabolic syndrome. Once activated, NLRP3 nucleates the assembly of the inflammasome complex and promotes caspase-1–mediated cleavage of pro-IL-1β and pro-IL-18 into their biologically active forms, driving pyroptotic cell death and amplifying the local inflammatory cascade in periodontal tissues. NLRP3-derived IL-1β potently upregulates RANKL expression in osteoblasts and PDL cells, directly linking inflammasome activation to alveolar bone resorption [14]. Endocrine mediators modulate NLRP3 activity in multiple ways: vitamin D signaling suppresses NLRP3 transcription through VDR-mediated mechanisms; glucocorticoids at physiological concentrations inhibit inflammasome priming by reducing NF-κB-driven NLRP3 transcription, whereas glucocorticoid excess or receptor resistance may paradoxically permit unopposed NLRP3 activity; insulin resistance promotes NLRP3 activation through ROS generation and AGE-RAGE signaling; and thyroid dysfunction, through its effects on mitochondrial function and oxidative metabolism, creates a cellular environment permissive for NLRP3 priming [15]. Emerging pharmacological approaches targeting inflammasome signaling, including NLRP3 inhibitors such as MCC950, IL-1 receptor antagonists, and caspase-1 inhibitors, are currently under preclinical investigation for both metabolic and inflammatory conditions, and their relevance to periodontal host-modulation therapy represents a promising future direction discussed further in Section 11.

Oxidative stress, generated by hyperglycemia-driven mitochondrial electron transport chain uncoupling, xanthine oxidase activation, and NADPH oxidase (NOX) induction in periodontal cells, acts as a convergent upstream activator of both NF-κB and NLRP3 inflammasome signaling. ROS oxidize IκBα, facilitating its proteasomal degradation and allowing NF-κB nuclear translocation, while simultaneously providing the second signal required for NLRP3 inflammasome assembly following TLR-mediated priming [16, 17]. This ROS-NF-κB-NLRP3 triad constitutes a core mechanistic cascade through which multiple endocrine perturbations—including insulin resistance, hypoestrogenism, glucocorticoid dysregulation, and vitamin D deficiency—converge to amplify periodontal inflammatory responses beyond the threshold of homeostatic containment [18].

Crucially, the relationship between periodontitis and endocrine disorders appears to be bidirectional: not only do hormonal imbalances predispose individuals to more severe and progressive periodontitis, but the chronic inflammatory state sustained by periodontal disease can in turn disrupt endocrine function through the systemic dissemination of pro-inflammatory mediators, as examined in detail in Section 10 [3, 8, 9].

This bidirectionality has important clinical implications, as it suggests that managing periodontal inflammation may contribute to improving endocrine homeostasis, just as better hormonal control may protect periodontal health. Evidence from Mendelian randomization studies is beginning to provide causal support for several of these associations, moving the field beyond purely epidemiological inference [19].

Despite growing recognition of the links between oral and endocrine health, the specific molecular pathways mediating this bidirectional relationship remain incompletely understood and have not been comprehensively synthesized. Most available reviews focus narrowly on a single hormonal axis—most commonly the sex hormone or insulin axes—without integrating the broader molecular network perspective or the evidence emerging from systems-level analyses. The present review aims to address this gap by providing a comprehensive, pathway-centered analysis of how hormonal imbalance and periodontal disease intersect at the molecular level.

Drawing on our prior characterization of the periodontitis diseasome and its inflammatory mediator networks [3, 4, 8], we systematically examine the molecular mechanisms through which key endocrine conditions—including sex steroid imbalances, metabolic hormone dysregulation, thyroid dysfunction, glucocorticoid excess or deficiency, and altered mineral-regulating hormone signaling—contribute to periodontal pathogenesis, and how periodontal inflammation in turn modulates endocrine function. Our central objective is to delineate the shared molecular pathways that mediate this bidirectional crosstalk, with the ultimate goal of informing integrated diagnostic and therapeutic strategies for patients in whom oral and endocrine health are simultaneously compromised.

The present review’s primary novel contribution is the integration of multiple endocrine axes—previously examined in isolation in existing reviews—into a unified, pathway-centered molecular framework anchored by the RANKL/OPG axis as a convergence point and informed by systems-level diseasome analysis. Unlike existing reviews that focus on a single hormonal system (e.g., diabetes-periodontitis, sex hormones, vitamin D), we offer a multi-axis synthesis that reveals common signaling hubs (TNF, IL6, STAT3, LEP, NOS3), shared pathways (PI3K/Akt, AGE-RAGE, JAK-STAT, Th17 differentiation, HIF-1), and the bidirectional nature of the endocrine-periodontal relationship as a whole. We recognize that clinical recommendations derived from this framework must be calibrated to the strength of available evidence, and we explicitly stratify evidence levels throughout the manuscript and in a dedicated summary table.

### 1.1 Methods

This narrative review was conducted through a structured, non-systematic literature search of PubMed/MEDLINE, Web of Science, Scopus, and the DisGeNET, OMIM, and KEGG pathway databases, covering publications from January 2000 through May 2026, with particular emphasis on studies published between 2018 and 2026. Search terms included combinations of ‘periodontal disease,’ ‘periodontitis,’ ‘endocrine,’ ‘hormone,’ ‘insulin resistance,’ ‘diabetes,’ ‘adipokines,’ ‘thyroid,’ ‘glucocorticoids,’ ‘parathyroid hormone,’ ‘vitamin D,’ ‘RANKL/OPG,’ ‘NF-κB,’ ‘molecular pathway,’ ‘comorbidity network,’ and ‘diseasome,’ applied individually and in Boolean combinations.

Priority was given to (1) randomized controlled trials and meta-analyses addressing clinical endocrine-periodontal associations; (2) Mendelian randomization studies providing genetic causal inference; (3) molecular and mechanistic studies (*in vitro* and animal models) characterizing receptor-mediated endocrine effects in periodontal cells; and (4) systems biology and bioinformatics analyses of gene-disease associations. Relevant reviews and landmark studies predating 2018 were included where they provided foundational mechanistic context. The scope of the review encompasses pathological endocrine disorders, physiological hormonal transitions (puberty, pregnancy, menopause), and pharmacological hormone exposures. The present review does not aim to be exhaustive but rather to synthesize the most mechanistically informative and clinically relevant evidence across multiple endocrine axes.

#### 1.1.1 Abbreviations

Given the relatively large number of names and concepts discussed (in particular those used in the various figures), we will introduce all full names and abbreviations for reference in Glossary.

## 2 Hormones as modulators of periodontal tissue homeostasis

The periodontium is not merely a passive structural scaffold supporting the dentition; it is a hormonally responsive tissue whose cellular constituents express a diverse array of hormone receptors that render it directly susceptible to fluctuations in systemic endocrine signaling [11, 20, 21]. Estrogen receptors (ERα and ERβ) have been identified in gingival fibroblasts, periodontal ligament (PDL) cells, osteoblasts, and oral epithelial cells, where they mediate genomic and non-genomic signaling cascades that regulate collagen synthesis, extracellular matrix (ECM) remodeling, and local immune responses [12, 22, 23]. Androgen receptors (ARs) are similarly expressed in gingival tissue and PDL cells, where they modulate fibroblast proliferation and the production of inflammatory mediators [24–26]. Glucocorticoid receptors (GRs), present in virtually all periodontal cell types, transduce signals from both endogenous cortisol and exogenous corticosteroids, regulating the transcription of genes involved in inflammation resolution, tissue repair, and bone metabolism [27, 28]. The co-expression of multiple receptor classes within the same periodontal cell populations implies that hormonal signals do not act in isolation but interact in complex, context-dependent ways to collectively shape the inflammatory and regenerative state of the periodontal microenvironment [3].

Beyond the steroid hormone receptors, the periodontium expresses receptors for metabolic and peptide hormones that link systemic energy homeostasis to local tissue health. Insulin receptors and components of the insulin signaling cascade (including IRS-1 and phosphoinositide 3-kinase (PI3K)) have been detected in gingival fibroblasts and PDL cells, establishing a direct molecular link between systemic glycemic status and periodontal tissue function [29–32]. Leptin receptors (LEP-R), signaling through the JAK2 (Janus Kinase 2)/STAT3 pathway, are expressed in gingival tissue and PDL cells, where LEP stimulates the production of pro-inflammatory cytokines and matrix-degrading enzymes [33–35]. Adiponectin receptors (AdipoR1 and AdipoR2) are also present in PDL fibroblasts and exert opposing, anti-inflammatory effects by activating AMP-activated protein kinase (AMPK) and suppressing NF-κB translocation [36–38]. The presence of these metabolic hormone receptors within the periodontium means that conditions such as obesity, insulin resistance, and metabolic syndrome (characterized by dysregulated adipokine and insulin signaling) are capable of directly altering the molecular environment of periodontal tissues independently of, and in addition to, their well-characterized effects on systemic inflammation [3, 39].

Thyroid hormone receptors (TRα and TRβ) and the VDR, a nuclear receptor with broad immunomodulatory functions, further expand the endocrine responsiveness of the periodontium. Thyroid hormones regulate the metabolic activity and proliferative capacity of PDL fibroblasts and influence osteoblast differentiation, with both hypothyroid and hyperthyroid states capable of perturbing alveolar bone turnover through distinct but complementary mechanisms [40–42]. The VDR is expressed in gingival keratinocytes, fibroblasts, macrophages, and dendritic cells, where its activation by 1,25-dihydroxyvitamin D${}_{3}$ suppresses the production of pro-inflammatory cytokines, enhances antimicrobial peptide synthesis, and modulates the RANKL/OPG ratio governing osteoclastogenesis [43, 44]. Notably, VDR polymorphisms have been previously identified as genetic susceptibility factors for periodontitis, underscoring the functional importance of vitamin D signaling in periodontal defense [45, 46]. Parathyroid hormone (PTH) receptors expressed on osteoblasts and PDL cells participate in the regulation of alveolar bone remodeling, and dysregulation of the PTH axis—as occurs in secondary hyperparathyroidism associated with chronic kidney disease or vitamin D deficiency—can accelerate bone resorption in the periodontium through RANKL-dependent pathways [47–49].

The functional consequence of this broad receptor landscape is that the periodontium operates as an endocrine target organ, integrating hormonal signals alongside microbial and immune inputs to maintain tissue homeostasis. Under physiological conditions, this hormonal modulation is beneficial: estrogens promote collagen synthesis and support alveolar bone density; androgens regulate fibroblast activity and temper certain inflammatory cascades; glucocorticoids facilitate the resolution of acute inflammation; insulin promotes cell survival and tissue repair; vitamin D and thyroid hormones calibrate immune responsiveness and metabolic activity within the tissue. These signals collectively maintain a balance between the constant microbial challenge at the dentogingival interface and the host’s capacity to contain it without causing collateral tissue destruction [50]. The periodontium’s homeostatic equilibrium is thus not solely determined by the local microbial load or the intrinsic competence of the immune response, but is continuously co-regulated by the hormonal milieu in which periodontal cells operate [51].

When endocrine homeostasis is disrupted—whether by pathological conditions such as diabetes, thyroid disease, or adrenal dysfunction, by physiological transitions such as puberty, pregnancy, or menopause, or by pharmacological interventions including corticosteroid therapy or hormonal contraception, the hormonal inputs to periodontal cells are correspondingly altered, shifting the tissue toward a pro-inflammatory, pro-resorptive, or hypo-regenerative state [12, 52, 53]. These hormonal perturbations do not simply add to existing periodontal risk factors; they interact with immune and microbial variables in non-linear ways, amplifying or attenuating the tissue response to bacterial challenge. For instance, the reduction in estrogen during menopause not only decreases alveolar bone density directly through osteoclast activation, but also upregulates the local production of IL-1β, IL-6, and TNF-α in periodontal tissues, effectively lowering the inflammatory threshold at which microbiota-driven pathology can escalate [54, 55]. Understanding these receptor-mediated mechanisms is therefore fundamental to appreciating why endocrine imbalance so consistently emerges as a modifying factor in periodontal disease susceptibility and progression, and why a molecular pathway perspective is essential for characterizing this relationship in its full complexity.

## 3 Sex steroids and periodontal inflammation

### 3.1 Estrogens

Estrogens exert their effects on periodontal tissues through two distinct but complementary signaling mechanisms. The classical genomic pathway involves the diffusion of estradiol across the plasma membrane, where it binds to intracellular ERα or ERβ, inducing receptor dimerization and translocation to the nucleus [12]. These dimers bind to estrogen response elements (EREs) in the promoter regions of target genes, recruiting coactivators or corepressors to modulate transcription in a cell-type- and context-dependent manner. In gingival fibroblasts and PDL cells, this pathway upregulates the synthesis of ECM components including type I collagen and fibronectin, thereby supporting the structural integrity and mechanical resilience of the periodontium. Conversely, estrogen signaling also suppresses the transcription of several MMPs, particularly MMP-1, MMP-3, and MMP-8, which are the principal enzymes responsible for collagen degradation in inflamed periodontal tissues [11, 56].

In parallel, the non-genomic pathway operates through membrane-associated ERs that activate second messenger cascades—including the mitogen-activated protein kinase (MAPK), PI3K/Akt, and protein kinase C pathways—within minutes of hormone binding, modulating cytoskeletal dynamics, cell proliferation, and the rapid suppression of pro-inflammatory signaling in periodontal cells. Together, these dual mechanisms position estrogen as a pleiotropic regulator of periodontal tissue anabolism and inflammation control. The effects of estrogen on bone-forming and bone-resorbing cells within the alveolar periodontium are particularly consequential for disease susceptibility [56]. In osteoblasts, estrogen signaling promotes cell survival, enhances the secretion of OPG—the decoy receptor that competitively inhibits RANKL-mediated osteoclast differentiation—and stimulates the expression of bone morphogenetic proteins that support alveolar crest maintenance. In osteoclasts and their precursors, estrogen suppresses proliferation and accelerates apoptosis, while simultaneously reducing the RANKL/OPG ratio that governs net bone resorptive activity. In PDL cells, estrogen upregulates osteogenic differentiation markers including runt-related transcription factor 2 (RUNX2) and alkaline phosphatase, promoting the regenerative capacity of this tissue under inflammatory conditions [57, 58]. These cellular effects collectively explain why estrogen-sufficient states are associated with greater alveolar bone density and reduced clinical attachment loss, and why estrogen deficiency—whether physiological or pathological—predisposes to accelerated periodontal bone resorption through RANKL-dependent mechanisms that synergize with those already activated by IL-1β, IL-6, and TNF-α in the context of established periodontitis [12].

The hormonal transitions of the female reproductive lifespan define distinct windows of periodontal vulnerability. During the menstrual cycle, mid-cycle and luteal phase elevations in estrogen and progesterone are associated with transient increases in gingival vascularity, permeability, and inflammatory cell infiltration, phenomena that are exaggerated in the presence of pre-existing subgingival biofilm. During pregnancy, sustained high levels of estrogen (alongside progesterone) profoundly alter local immune responses in the gingival tissues, suppressing certain protective T-cell functions while amplifying prostaglandin E${}_{2}$ (PGE${}_{2}$) production and vascular permeability, contributing to the clinical entity known as pregnancy gingivitis, which affects a substantial proportion of pregnant women and can progress to periodontitis in susceptible individuals [59].

Critically, as previously documented, the systemic dissemination of periodontal pro-inflammatory mediators during pregnancy can reach the uteroplacental unit, where they may stimulate uterine contractions and promote preterm labor through mechanisms involving IL-1β, PGE${}_{2}$, and TNF-α, establishing periodontitis as a meaningful contributor to adverse obstetric outcomes [3].

The menopausal transition, characterized by a sustained and irreversible decline in ovarian estrogen production, represents perhaps the most clinically significant endocrine window for periodontal health: postmenopausal women exhibit significantly reduced alveolar bone density, increased clinical attachment loss, and higher susceptibility to periodontitis progression compared to premenopausal women, an effect that is partially mitigated by hormone replacement therapy (HRT), though the benefits of HRT must be weighed against its systemic risks. Oxidative stress constitutes an important molecular denominator linking hypoestrogenism to periodontal disease progression. Estrogen, through ERα-dependent upregulation of antioxidant enzymes including superoxide dismutase 2 (SOD2), catalase, and glutathione peroxidase, normally restrains the intracellular accumulation of reactive oxygen species (ROS) in periodontal cells [60]. Upon estrogen withdrawal, this antioxidant protection is diminished, leading to elevated ROS levels that activate redox-sensitive transcription factors (especially NF-κB) thereby amplifying the transcription of IL-1β, IL-6, TNF-α, and MMP genes in gingival fibroblasts and macrophages. This oxidative amplification of periodontal inflammation mirrors the oxidative stress mechanisms previously identified in the context of cardiovascular comorbidities of periodontitis [3], and underscores the importance of estrogen not merely as a bone-protective hormone but as a systemic modulator of inflammatory redox homeostasis. The convergence of reduced antioxidant capacity, elevated NF-κB activity, and impaired OPG secretion in hypoestrogenic periodontal tissues creates a molecular environment that is permissive for the transition from subclinical inflammation to overt, progressive periodontitis, even in the absence of significant changes in the subgingival microbiota [12, 61].

### 3.2 Progesterone

Progesterone receptors (PRs), expressed in gingival fibroblasts, vascular endothelial cells, and periodontal immune cells, mediate effects that are both complementary to and distinct from those of estrogen in the periodontium. Unlike estrogen, which generally exerts anti-inflammatory and tissue-anabolic effects at physiological concentrations, progesterone at elevated levels—as occur during the luteal phase of the menstrual cycle, throughout pregnancy, and in women using certain hormonal contraceptives—tends to amplify several components of the local inflammatory response in gingival tissues. A principal mechanism involves progesterone-mediated enhancement of arachidonic acid metabolism in gingival cells, leading to increased synthesis and release of PGE${}_{2}$, a potent mediator of vascular dilation, increased permeability, and osteoclast activation. Progesterone also upregulates the expression of MMP-1 and MMP-3 in gingival fibroblasts and suppresses the synthesis of fibroblast-derived collagen, effectively tilting the ECM remodeling balance toward degradation rather than repair [62]. These pro-resorptive and pro-inflammatory effects of progesterone on periodontal tissues are typically subclinical in women with good oral hygiene, but in the presence of microbial biofilm-driven inflammation, they act as powerful amplifiers of gingival and periodontal pathology. The clinical corollary is the well-recognized phenomenon of pregnancy-associated gingivitis, which typically manifests from the second trimester onward as biofilm mass remains relatively stable but the hormonal milieu dramatically increases tissue responsiveness to microbial challenge [63].

The progesterone-mediated amplification of periodontal inflammation has important implications for understanding the oral health effects of exogenous hormonal exposure. Older generations of combined oral contraceptives containing high progestogen doses were associated with significant gingival inflammation and increased bleeding on probing; contemporary low-dose formulations produce more attenuated effects, though mild increases in gingival inflammatory response compared to non-users persist in some studies [64]. Beyond contraception, progestogen-containing intrauterine devices, hormone replacement therapies combining estrogen with synthetic progestogens, and progesterone supplementation used in assisted reproduction protocols all represent clinical scenarios in which progesterone signaling may modulate the periodontal inflammatory environment. The molecular basis of these effects involves progesterone’s capacity to alter the sensitivity of gingival immune cells to bacterial LPS, including that of key periodontal pathogens such as *Porphyromonas gingivalis*, by modifying Toll-like receptor (TLR) signaling thresholds and enhancing the transcription of IL-1β and PGE${}_{2}$ biosynthetic enzymes. These interactions between exogenous progestogens and periodontal innate immunity underscore the need for clinicians in both dental and obstetric/gynecological settings to recognize hormonal status as a dynamic modifier of periodontal disease susceptibility.

### 3.3 Androgens

Androgen receptors (ARs), activated principally by testosterone and its more potent metabolite dihydrotestosterone (DHT), are expressed in gingival fibroblasts, PDL cells, and periodontal immune cells, where they participate in the regulation of inflammatory gene expression, cell proliferation, and ECM metabolism. At physiological concentrations, androgen signaling exerts generally modulatory effects on periodontal inflammation [65]: ARs activation has been shown to suppress NF-κB transcriptional activity in gingival fibroblasts, thereby reducing the basal expression of IL-6, IL-8, and TNF-α, three of the most prominently elevated cytokines in periodontitis and hub molecules in the periodontitis molecular comorbidity network [4]. This NF-κB–suppressive activity of androgens parallels mechanisms documented in other inflammatory tissues and suggests that physiological androgen levels may constitute a degree of endogenous anti-inflammatory protection in the periodontium. Epidemiological data are consistent with this interpretation: men generally exhibit higher periodontal attachment loss and greater tooth loss than women of comparable age, an observation traditionally attributed to differences in health behaviors, but increasingly recognized as also reflecting hormonal influences on inflammatory susceptibility [21, 61]. The relationship between testosterone decline in aging men—analogous to the estrogenic decline of menopause—and increasing periodontal disease severity further supports a modulatory role of androgens in long-term periodontal homeostasis.

The endocrine condition most directly linking androgen excess to periodontal pathology in women is polycystic ovary syndrome (PCOS), a highly prevalent endocrine disorder characterized by hyperandrogenism, oligo-anovulation, and often insulin resistance. Women with PCOS exhibit significantly higher rates of periodontitis and more severe clinical parameters, including greater probing depth, clinical attachment loss, and gingival bleeding—compared to age, and body mass index–matched controls without the syndrome [66]. At the molecular level, the hyperandrogenic and insulin-resistant environment of PCOS creates a compounded risk: elevated androgens may paradoxically promote a pro-inflammatory phenotype in the context of concurrent insulin resistance, where the NF-κB–suppressive capacity of ARs signaling appears to be overridden by insulin resistance–associated upregulation of inflammatory cascades. PCOS-associated elevations in circulating TNF-α, IL-6, and C-reactive protein (CRP) mirror the systemic inflammatory signature of periodontitis and share the same hub gene connectivity—particularly through STAT3 and IL6—identified in the periodontitis diseasome [4, 66], suggesting that the bidirectional molecular crosstalk between periodontal inflammation and PCOS operates through converging inflammatory and metabolic signaling nodes rather than exclusively through androgen receptor pathways. These shared molecular origins raise the prospect that periodontal treatment may contribute to reducing systemic inflammatory burden in women with PCOS, a hypothesis that warrants dedicated clinical investigation.

An emerging and clinically important dimension of androgen–periodontal interactions concerns gender-affirming hormone therapy (GAHT) in transgender individuals. Transgender women (assigned male at birth) undergoing feminizing GAHT, which typically involves the administration of exogenous estrogens combined with androgen-deprivation agents, experience a progressive shift in their hormonal milieu toward an estrogenic profile. Conversely, transgender men (assigned female at birth) receiving masculinizing GAHT with exogenous testosterone transition toward higher androgenic tone [59]. These pharmacologically induced hormonal transitions provide a unique natural experiment for studying the effects of sex steroids on periodontal tissues in humans. Emerging data suggest that transgender women undergoing feminizing therapy may experience changes in gingival inflammatory responses consistent with the known effects of estrogen on periodontal vascularity and immune modulation, while transgender men receiving testosterone may demonstrate alterations in gingival fibroblast behavior and cytokine profiles. Although the evidence base remains limited and methodologically heterogeneous, these observations are consistent with the receptor-mediated mechanisms described above and underscore the clinical importance of tailoring periodontal surveillance to the hormonal context of each individual patient, irrespective of biological sex assigned at birth [67].

## 4 Metabolic hormones: insulin, adipokines, and the periodontal-metabolic axis

### 4.1 Insulin and insulin resistance

Insulin signaling in periodontal tissues operates through a well-defined molecular cascade whose disruption under conditions of insulin resistance generates a pro-inflammatory, pro-resorptive environment directly conducive to periodontitis progression. Under physiological conditions, insulin binding to its receptor in gingival fibroblasts and PDL cells activates IRS-1–mediated PI3K/Akt signaling, promoting cell survival, glucose uptake, collagen synthesis, and the suppression of pro-inflammatory gene transcription. In states of chronic hyperinsulinemia and insulin resistance (such as in type 2 diabetes mellitus, obesity, and metabolic syndrome) this protective signaling is impaired through at least two converging mechanisms. First, elevated circulating concentrations of pro-inflammatory cytokines, including TNF-α and IL-6, induce the expression of SOCS-3 in periodontal cells, which directly phosphorylates and targets IRS-1 for proteasomal degradation, severing the insulin receptor from its downstream anabolic and anti-inflammatory effectors. Second, the chronic hyperglycemia associated with inadequate glycemic control drives the non-enzymatic glycation of periodontal proteins, generating AGEs that bind to RAGE on gingival macrophages, endothelial cells, and fibroblasts, triggering sustained NF-κB activation and the transcription of IL-1β, IL-6, TNF-α, and MMP-8, the precise inflammatory signature that characterizes active periodontal lesions [68, 69]. The AGE-RAGE axis thus constitutes a molecular transducer that converts systemic glycemic dysregulation into amplified local periodontal inflammation, explaining at the mechanistic level why poorly controlled diabetes is consistently associated with higher periodontal disease prevalence, greater attachment loss, and faster disease progression than normoglycemic states.

The AGE-RAGE axis activates NLRP3 inflammasome assembly in periodontal macrophages and gingival fibroblasts, generating a burst of IL-1β and IL-18 that amplifies NF-κB-driven cytokine transcription in a self-reinforcing loop, and that directly upregulates RANKL in osteoblasts, compounding the osteoclastogenic stimulus already present in insulin-resistant periodontal tissues [15].

The bidirectional relationship between insulin resistance and periodontitis—previously characterized in detail from an inflammatory and comorbidity perspective [3, 4, 70], acquires additional mechanistic depth when examined through the lens of hormonal signaling. Chronic periodontal inflammation disrupts insulin sensitivity through several interconnected pathways: the systemic spillover of periodontal-derived IL-1β, IL-6, and TNF-α induces hepatic and peripheral SOCS-3 expression, impairing insulin receptor signaling in tissues far removed from the oral cavity; LPS released from periodontal pathogens such as *P. gingivalis* activates TLR4 on adipocytes and skeletal muscle cells, promoting serine phosphorylation of IRS-1 and further uncoupling insulin receptor activation from its metabolic effectors; and periodontal bacteremia-driven increases in circulating acute-phase reactants, particularly CRP and fibrinogen, directly interfere with insulin receptor substrate function at the cellular level. The net result is a self-reinforcing cycle in which insulin resistance exacerbates periodontal inflammation through AGE-RAGE and NF-κB–mediated mechanisms, while periodontal inflammation worsens insulin resistance through cytokine- and LPS-mediated IRS-1 disruption [68]. This molecular reciprocity provides a mechanistic foundation for the clinically observed improvements in glycated hemoglobin (HbA1c) following successful periodontal therapy in patients with type 2 diabetes, a finding that positions periodontal treatment not merely as oral healthcare but as a meaningful adjunct to metabolic disease management.

### 4.2 Adipokines

Adipokines—hormone-like proteins secreted by adipose tissue that coordinate metabolic, immune, and inflammatory functions—constitute a molecular interface of exceptional importance between systemic endocrine dysregulation and periodontal pathology. Among these, LEP and adiponectin are the most extensively studied in the periodontal context and exhibit sharply opposing functional profiles. Leptin, acting through its receptor LEP-R via the JAK2/STAT3 signaling axis, promotes a pro-inflammatory phenotype in periodontal cells: it upregulates the production of TNF-α, IL-6, and IL-12 in gingival macrophages; synergistically enhances MMP-1 and MMP-3 secretion by gingival fibroblasts in response to bacterial stimulation; and activates osteoclast differentiation through RANKL upregulation [71].

Critically, the gene encoding LEP was identified as one of the principal hub genes in the periodontitis molecular comorbidity network, with a connectivity degree spanning 11 distinct disease conditions including obesity, type 2 diabetes, depressive disorder, and non-alcoholic fatty liver disease [4] illustrating that LEP’s role in periodontitis is not an isolated phenomenon but part of a broader inflammatory molecular network shared across metabolic and psychiatric comorbidities. Consistent with this, salivary and serum LEP levels are significantly elevated in patients with periodontitis compared to periodontally healthy controls, correlate positively with clinical parameters of disease severity including probing depth and clinical attachment loss, and decrease following successful non-surgical periodontal therapy, validating LEP as both a mechanistic contributor and a clinically informative biomarker of periodontal inflammation [72].

Adiponectin exerts a strikingly contrasting set of effects that position it as a molecular guardian of periodontal homeostasis whose insufficiency (as characteristically occurs in obesity, type 2 diabetes, and metabolic syndrome) amplifies periodontal susceptibility. Adiponectin signals through AdipoR1 and AdipoR2 receptors expressed on PDL fibroblasts, gingival macrophages, and osteoblasts, activating AMPK and Peroxisome proliferator-activated receptor-alpha (PPARα) pathways that collectively suppress NF-κB nuclear translocation and reduce the transcription of IL-1β, IL-6, IL-8, MMP-1, and MMP-3. Simultaneously, adiponectin induces the production of the anti-inflammatory cytokine IL-10 and the cytoprotective enzyme heme oxygenase-1 (HO-1) in periodontal macrophages, shifting these cells toward a pro-resolution phenotype. In osteoclast precursors, adiponectin acts through a Forkhead box proteins O1 (FOXO1)-dependent mechanism to inhibit differentiation and promote apoptosis, thereby restraining alveolar bone resorption independently of the RANKL/OPG axis [73].

In osteoblasts and PDL cells, adiponectin upregulates RUNX2 expression and promotes the synthesis of OPG, further protecting alveolar bone integrity. Serum adiponectin levels are consistently reduced in patients with periodontitis compared to healthy controls and decrease further in the context of co-existing obesity or diabetes; conversely, successful periodontal therapy is associated with significant restoration of circulating adiponectin levels, suggesting that controlling oral inflammation can partially reverse the adipokine dysregulation of metabolic disease [73]. Beyond LEP and adiponectin, visfatin (also known as NAMPT, nicotinamide phosphoribosyltransferase) has emerged as a pro-inflammatory adipokine whose enzymatic activity generates the NAD${}^{+}$ precursor nicotinamide mononucleotide while simultaneously activating NF-κB and stimulating the production of IL-1β and IL-6 in PDL fibroblasts; elevated visfatin levels have been detected in the gingival crevicular fluid (GCF) of periodontitis patients, where they correlate with local inflammatory burden. Resistin and omentin-1 represent additional adipokines with emerging relevance as GCF biomarkers: resistin promotes pro-inflammatory macrophage polarization and has been proposed as a potential periodontal disease biomarker through systematic meta-analysis, while omentin-1 exerts insulin-sensitizing and anti-inflammatory effects whose reduction in obesity may contribute to periodontal susceptibility. Together, these adipokines constitute a hormonally driven molecular layer that continuously modulates the inflammatory tone of the periodontium in response to the metabolic state of the organism [74–76].

### 4.3 Glucagon-like peptide-1 (GLP-1) and incretins

Glucagon-like peptide-1 (GLP-1) is an incretin hormone secreted by intestinal L-cells in response to nutrient ingestion, where it potentiates glucose-stimulated insulin secretion, suppresses glucagon release, and delays gastric emptying to support postprandial glycemic control. Beyond its canonical metabolic functions, GLP-1 exerts pleiotropic anti-inflammatory and cytoprotective effects through its receptor (GLP-1R), which is expressed not only in pancreatic beta cells but also in macrophages, endothelial cells, osteoblasts, and—critically for the present discussion—in cells of the periodontium including gingival fibroblasts and PDL cells [77].

GLP-1R activation suppresses NF-κB signaling and reduces the transcription of TNF-α, IL-6, and IL-1β in macrophages through a Cyclic adenosine monophosphate (cAMP)/Protein kinase A (PKA)–dependent mechanism, promotes osteoblast differentiation and survival while inhibiting osteoclastogenesis through RANKL-independent pathways, and enhances the synthesis of collagen and growth factors involved in connective tissue repair. The clinical explosion of GLP-1 receptor agonists (GLP-1RAs), including semaglutide, liraglutide, and dulaglutide, now prescribed at unprecedented scale for type 2 diabetes and obesity management, has created an urgent and clinically timely question: do these agents exert beneficial effects on periodontal health through their direct anti-inflammatory and bone-protective mechanisms, independent of their glycemic and weight-reducing effects? Preliminary experimental evidence is encouraging: animal studies demonstrate that GLP-1RA administration reduces alveolar bone loss in ligature-induced periodontitis models, attenuates gingival inflammatory cell infiltration, and lowers local concentrations of IL-1β and MMP-8; *in vitro* studies show that GLP-1R activation in human PDL fibroblasts suppresses LPS-induced cytokine production and promotes osteogenic differentiation markers [78, 79].

Clinical data in humans remain sparse and mostly observational, but patients with type 2 diabetes treated with GLP-1RAs show trends toward reduced periodontal inflammatory indices compared to those managed with other antidiabetic agents, a difference that appears to persist after adjustment for glycemic control, suggesting a direct periodontal effect beyond glucose lowering. As GLP-1RAs increasingly enter the management of non-diabetic obesity and are being explored for cardiovascular and renal protection, understanding their impact on the periodontal inflammatory environment represents both a scientific priority and a practical clinical consideration, given the high prevalence of periodontitis in the patient populations most likely to receive these medications.

## 5 Thyroid hormones and periodontal disease

Thyroid hormones, principally triiodothyronine (T3) and thyroxine (T4), are master regulators of cellular metabolism, proliferation, and differentiation across virtually all tissues in the organism, and the periodontium is no exception [80]. Thyroid hormone receptors TRα and TRβ, members of the nuclear receptor superfamily, are expressed in gingival fibroblasts, PDL cells, osteoblasts, and periodontal epithelial cells, where they function as ligand-activated transcription factors that regulate the expression of genes involved in ECM synthesis, cell cycle progression, and mitochondrial biogenesis. Upon binding T3, these receptors interact with thyroid hormone response elements (TREs) in the promoter regions of target genes, modulating the transcriptional programs that govern fibroblast metabolic activity, collagen turnover rate, and the regenerative capacity of periodontal connective tissues [81].

In osteoblasts, T3 signaling directly stimulates the expression of alkaline phosphatase, osteocalcin, and RUNX2, promoting bone matrix synthesis and mineralization, while simultaneously upregulating RANKL expression in a dose-dependent fashion, such that the net effect of thyroid hormone on alveolar bone depends critically on the magnitude and chronicity of hormonal exposure [82–84]. These receptor-mediated effects establish that the periodontal cellular machinery is continuously calibrated by thyroid hormone tone, and that deviations from euthyroid status in either direction carry distinct and clinically meaningful consequences for periodontal tissue homeostasis.

Hypothyroidism, characterized by insufficient production of T3 and T4, impairs periodontal health through multiple converging mechanisms that collectively reduce the regenerative and immunological competence of the tissue [41, 42, 85]. In states of thyroid hormone deficiency, gingival fibroblast metabolic activity is diminished [86–88], resulting in reduced collagen synthesis rates, impaired ECM turnover, and delayed wound healing responses that compromise the capacity of periodontal tissues to recover from inflammatory insults. The reduced basal metabolic rate of hypothyroid cells is accompanied by diminished mitochondrial function and increased susceptibility to oxidative stress, creating a cellular environment in which ROS accumulation amplifies NF-κB activity and pro-inflammatory cytokine production even in the absence of overt microbial challenge [89]. Osteoblast function is similarly impaired in hypothyroidism [90], with reduced expression of bone formation markers and a shift in the RANKL/OPG balance toward net bone resorption at the alveolar crest [91–93].

Clinically, patients with hypothyroidism exhibit higher rates of periodontitis, greater probing depth, and more pronounced clinical attachment loss compared to euthyroid controls, an association that has been supported by cohort data demonstrating that low thyroid-stimulating hormone (TSH) levels correlate with significantly elevated periodontitis prevalence [94–96]. Furthermore, the immunosuppressive effects of hypothyroidism, including reduced neutrophil chemotaxis and impaired phagocytic capacity, may blunt the early innate immune response to periodontal pathogens, allowing microbial dysbiosis to become established more readily and with less effective containment [40].

Hyperthyroidism presents a contrasting but equally detrimental profile for periodontal health, primarily through the acceleration of bone metabolic turnover beyond the capacity for adequate mineralization and structural consolidation [41]. Excess T3 drives osteoclast activity through direct upregulation of RANKL in osteoblasts and indirect enhancement of IL-6 and IL-1β production in periodontal immune cells, resulting in accelerated alveolar bone resorption that is mechanistically independent of, though additive to, the bone loss driven by local periodontal inflammation [95]. The hypermetabolic state of hyperthyroidism also increases oxidative phosphorylation rates in periodontal cells, generating elevated ROS that activate redox-sensitive inflammatory pathways and further amplify the local inflammatory response to subgingival bacteria [97]. An additional mechanism of clinical relevance involves the TSH receptor itself: TSH has been shown to exert direct bone-protective effects through TSH receptor signaling on osteoblasts and osteoclasts that are independent of its role in regulating thyroid hormone synthesis, such that the suppressed TSH levels characteristic of hyperthyroidism represent not merely a diagnostic indicator but an active contributor to net bone loss through loss of this TSH-mediated protective axis. Patients with Graves disease, the most common cause of hyperthyroidism, exhibit periodontal inflammatory indices including elevated GCF levels of IL-6 and TNF-α that correlate with both disease severity and degree of thyroid hormone excess, suggesting that the inflammatory signatures of Graves disease and periodontitis converge at shared molecular nodes consistent with the cytokine hub architecture previously characterized in the periodontitis diseasome [4].

In both hypothyroid and hyperthyroid states, the mitochondrial dysfunction and elevated ROS generation that accompany thyroid hormone imbalance create the bioenergetic conditions that prime NLRP3 inflammasome activation, thereby contributing to the elevated IL-1β and IL-18 detected in the GCF of patients with active periodontitis and concurrent thyroid disease [85].

Beyond the classical actions of T3 and T4, thyroid autoimmune conditions introduce an additional immunological dimension to the thyroid-periodontal relationship. Hashimoto thyroiditis, the most prevalent autoimmune thyroid disease and principal cause of hypothyroidism, is characterized by lymphocytic infiltration of the thyroid, circulating anti-thyroid peroxidase (anti-TPO) and anti-thyroglobulin antibodies, and a systemic Th1/Th17-skewed immune profile that closely mirrors the adaptive immune dysregulation documented in chronic periodontitis [98]. The shared immunological features between Hashimoto thyroiditis and periodontitis, including elevated circulating IL-17, reduced Treg/Th17 ratios, and aberrant B cell activation, suggest that individuals with one condition may carry an immune predisposition that facilitates the other [99]. Emerging bidirectional Mendelian randomization evidence supports a causal relationship between periodontal disease and thyroid dysfunction, indicating that the association is not solely explained by shared confounders such as smoking or socioeconomic status [40].

Periodontopathic bacteria, particularly *P. gingivalis*, may further contribute to thyroid autoimmunity through molecular mimicry mechanisms, in which bacterial protein epitopes share structural homology with thyroid antigens and thereby stimulate cross-reactive immune responses, a mechanism analogous to that proposed for the relationship between *P. gingivalis* and rheumatoid arthritis through citrullinated protein antigens [3].

The therapeutic implications of the thyroid-periodontal relationship are beginning to attract clinical and investigative attention, though the evidence base remains in early stages of development [94]. Thyroid hormone replacement with levothyroxine in hypothyroid patients has been associated with partial normalization of periodontal inflammatory parameters in observational studies, consistent with the restoration of fibroblast metabolic activity, improved neutrophil function, and re-establishment of a more favorable RANKL/OPG balance following euthyroid status [100]. Conversely, effective treatment of hyperthyroidism with antithyroid medications or radioiodine therapy has been linked to stabilization of alveolar bone loss parameters, reflecting the attenuation of T3-driven osteoclastogenesis and the recovery of TSH-mediated bone protection [85]. From a diagnostic standpoint, screening for thyroid dysfunction in patients presenting with refractory or rapidly progressive periodontitis may identify a subset of individuals in whom endocrine optimization would meaningfully improve periodontal treatment outcomes. The reverse proposition, that periodontal assessment should be incorporated into the routine clinical evaluation of patients with newly diagnosed thyroid disease, is equally warranted given the bidirectional nature of the relationship and the high prevalence of unrecognized periodontitis in populations with endocrine comorbidities [40].

Interdisciplinary collaboration between periodontists and endocrinologists, analogous to models already established for diabetes management, represents the most rational framework for translating the growing mechanistic understanding of this relationship into integrated patient care [4].

## 6 Glucocorticoids, the HPA axis, and periodontal susceptibility

The hypothalamic-pituitary-adrenal (HPA) axis, the principal neuroendocrine system governing the organism’s response to physiological and psychological stress, exerts pervasive regulatory effects on immune function, inflammation resolution, and tissue homeostasis through the actions of glucocorticoids, primarily cortisol in humans [101, 102]. Glucocorticoid receptors (GRs), belonging to the nuclear receptor superfamily, are expressed in virtually every periodontal cell type, including gingival epithelial cells, fibroblasts, macrophages, neutrophils, PDL cells, and osteoblasts, where they mediate both genomic and non-genomic effects on inflammatory gene transcription and cellular metabolism [28]. Upon cortisol binding, the GR translocates to the nucleus and interacts with glucocorticoid response elements (GREs) in the promoters of anti-inflammatory genes, inducing the transcription of lipocortin-1, IL-10, and the inhibitor of NF-κB (IκBα), while simultaneously engaging in transrepression interactions with NF-κB and activator protein 1 (AP-1) that suppress the transcription of IL-1β, IL-6, TNF-α, MMP-1, MMP-3, and cyclooxygenase-2 (COX-2). Under physiological conditions, this glucocorticoid-mediated immunomodulation performs a critical homeostatic function in the periodontium, restraining the amplitude and duration of the innate immune response to the constant microbial challenge at the dentogingival interface and facilitating the resolution of acute inflammatory episodes before they escalate to chronic tissue-destructive states [103–105]. The periodontium is thus continuously shaped by cortisol tone, and perturbations of the HPA axis in either direction carry significant consequences for the balance between periodontal protection and vulnerability [106–108].

Chronic psychological stress, now recognized as a significant modifier of periodontal disease risk and progression, exerts its pathological effects on the periodontium through sustained dysregulation of the HPA axis and its downstream glucocorticoid signaling. Prolonged psychosocial stress induces persistent cortisol hypersecretion followed, in many individuals, by HPA axis dysregulation characterized by blunted cortisol awakening responses, flattened diurnal cortisol rhythms, and altered glucocorticoid receptor sensitivity in immune cells [106]. This pattern of chronic HPA dysregulation has dual pathological consequences for periodontal health: on one hand, the sustained elevation of cortisol suppresses protective immune surveillance in the periodontium by impairing neutrophil chemotaxis, reducing secretory IgA levels in saliva, and attenuating the phagocytic capacity of gingival macrophages, thereby facilitating the unchecked expansion of dysbiotic subgingival biofilms; on the other hand, the glucocorticoid receptor resistance that develops in immune cells following prolonged cortisol exposure paradoxically allows NF-κB and AP-1 driven pro-inflammatory transcription to proceed with reduced GR-mediated transrepression, generating a state in which both immune protection and inflammatory regulation are simultaneously impaired [109, 110].

Epidemiological and clinical studies have consistently demonstrated associations between psychological stress, depression, and more severe periodontitis, with higher cortisol levels in GCF and saliva correlating with greater probing depth and attachment loss [111–114]. These associations are not confounded solely by stress-related behavioral changes such as poor oral hygiene or increased smoking, as they persist in multivariate analyses adjusting for these factors, suggesting that direct neuroendocrine effects on periodontal tissue homeostasis contribute independently to stress-associated periodontal vulnerability. Pathological states of glucocorticoid excess, whether of endogenous or exogenous origin, represent a more severe form of HPA-periodontal interaction with well-characterized clinical and molecular consequences. Endogenous hypercortisolism, as occurs in Cushing syndrome due to pituitary adenoma, adrenal tumors, or ectopic ACTH secretion, produces a constellation of systemic effects that converge on the periodontium to dramatically increase disease [111, 112].

The sustained supraphysiological cortisol levels of Cushing syndrome suppress osteoblast differentiation and promote osteoclastogenesis through multiple mechanisms including downregulation of Wnt/β-catenin signaling in osteoblast progenitors, direct inhibition of IGF-1 receptor signaling in PDL cells, and upregulation of RANKL expression in osteoblasts and T cells, resulting in accelerated alveolar bone loss that is additive to any microbiota-driven destruction [115–117].

Concurrently, glucocorticoid excess impairs the functional integrity of the gingival epithelial barrier by reducing the expression of tight junction proteins including claudin-1 and E-cadherin, increasing epithelial permeability to bacterial products and facilitating the translocation of periodontal pathogens and their endotoxins into the submucosa and bloodstream [118, 119]. The oral manifestations of Cushing syndrome, including gingival fragility, impaired wound healing, and increased susceptibility to oral infections, are direct clinical expressions of these molecular perturbations and warrant systematic periodontal evaluation as part of the endocrine workup of affected patients [120].

Exogenous glucocorticoid administration, representing one of the most widely prescribed pharmacological interventions in medicine for conditions ranging from autoimmune diseases to organ transplantation and chronic inflammatory disorders, generates equivalent or greater degrees of periodontal vulnerability depending on dose, duration, and route of administration. Long-term systemic corticosteroid therapy suppresses the HPA axis through negative feedback, produces iatrogenic Cushing syndrome at high doses, and profoundly impairs the periodontal immune response by reducing circulating neutrophil counts and function, impairing monocyte differentiation into macrophages, and suppressing the adaptive immune responses mediated by T and B lymphocytes that normally contain periodontal pathogen proliferation. At the molecular level, exogenous glucocorticoids further reduce the production of OPG by PDL fibroblasts and osteoblasts, shifting the RANKL/OPG ratio toward net osteoclastic activity even in the absence of significant local inflammation [121–123]. Patients receiving long-term corticosteroid therapy for conditions such as rheumatoid arthritis, inflammatory bowel disease, asthma, or following solid organ transplantation exhibit significantly higher rates of periodontitis, more pronounced alveolar bone loss on radiographic assessment, and poorer responses to conventional periodontal therapy compared to non-steroid-treated controls. Notably, several of these underlying conditions for which glucocorticoids are prescribed share molecular origins with periodontitis, as documented in the periodontitis comorbidity network [4], suggesting that the periodontal vulnerability of these patients reflects both the direct pharmacological effects of glucocorticoids and the pro-inflammatory molecular background of their underlying systemic disease.

Prolonged glucocorticoid receptor resistance in immune cells, as occurs following sustained cortisol exposure, may paradoxically release NLRP3 from glucocorticoid-mediated transcriptional suppression, enabling unopposed inflammasome activity in periodontal tissues despite elevated systemic cortisol concentrations—a mechanism that may partly explain the exaggerated periodontal inflammatory responses observed in chronic stress states [124, 125].

The opposite end of the glucocorticoid spectrum, adrenal insufficiency, presents a distinct but equally significant profile of periodontal risk. Primary adrenal insufficiency, known as Addison disease, is characterized by the destruction of adrenocortical tissue, typically through autoimmune mechanisms, resulting in deficiency of both cortisol and aldosterone. The loss of glucocorticoid anti-inflammatory regulation in Addison disease removes the physiological brake on NF-κB-driven cytokine production in periodontal tissues, potentially leading to exaggerated local inflammatory responses to bacterial challenge and accelerated tissue destruction, though the specific periodontal phenotype of untreated or inadequately replaced adrenal insufficiency remains incompletely characterized [126, 127]. The oral manifestations of Addison disease, including characteristic mucosal hyperpigmentation at sites of trauma such as the gingiva and buccal mucosa, serve as early diagnostic indicators that bring these patients to dental attention, creating an important opportunity for periodontal evaluation and interdisciplinary referral [128]. Secondary adrenal insufficiency, arising from prolonged exogenous corticosteroid therapy followed by abrupt discontinuation or inadequate tapering, may produce a state of relative glucocorticoid deficiency in which the blunted HPA response fails to generate sufficient cortisol to modulate periodontal inflammatory responses during infectious or surgical challenges. This consideration is clinically relevant for periodontal surgeons managing patients with a history of corticosteroid therapy, in whom adrenal supplementation protocols may be necessary to prevent exaggerated inflammatory responses and impaired healing following periodontal procedures.

Adrenal gland hypofunction was identified as a condition sharing associated genes with periodontitis in the molecular comorbidity network [4], providing database-supported molecular evidence for a biologically grounded relationship that clinical observations have long suggested but incompletely explained.

The bidirectional nature of the HPA-periodontal relationship deserves explicit recognition, as periodontal inflammation is not merely a consequence of glucocorticoid dysregulation but can itself alter HPA axis function through systemic inflammatory mechanisms. Chronically elevated circulating levels of IL-1β, IL-6, and TNF-α, as occur in severe and generalized periodontitis, are known activators of the HPA axis at the hypothalamic level, stimulating corticotropin-releasing hormone (CRH) and adrenocorticotropic hormone (ACTH) secretion and thereby elevating baseline cortisol production [129–131].

This inflammation-driven HPA activation, initially adaptive, may over time contribute to the cortisol dysregulation and receptor resistance patterns observed in patients with chronic inflammatory diseases. Furthermore, the neuroinflammatory consequences of periodontal bacteremia and cytokine spillover, previously documented in connection with cognitive decline and depression [3], may alter HPA axis setpoints through effects on hypothalamic nuclei and hippocampal glucocorticoid receptor expression, establishing a mechanistic pathway by which chronic oral inflammation contributes to the neuroendocrine dysfunction that further compromises periodontal and systemic health. This integrated perspective, in which the HPA axis simultaneously modulates and is modulated by periodontal inflammatory status, reinforces the case for incorporating stress assessment, cortisol evaluation, and adrenal function screening into the comprehensive management of patients with severe or treatment-refractory periodontitis.

## 7 Parathyroid hormone, vitamin D, and alveolar bone homeostasis

The maintenance of alveolar bone integrity is a central determinant of periodontal health, and the hormonal regulation of calcium and phosphate metabolism through the parathyroid hormone (PTH) and vitamin D axes constitutes one of the most direct endocrine interfaces with periodontal tissue homeostasis. PTH, a peptide hormone secreted by the chief cells of the parathyroid glands in response to declining serum ionized calcium concentrations, acts on bone, kidney, and intestine to restore calcium homeostasis through a coordinated set of cellular responses that include stimulation of osteoclastic bone resorption, enhancement of renal calcium reabsorption, and upregulation of renal 1-alpha-hydroxylase, the enzyme responsible for the conversion of 25-hydroxyvitamin D to its biologically active form, 1,25-dihydroxyvitamin D3 (calcitriol) [132–134].

PTH 1 receptor (PTH1R) is abundantly expressed on osteoblasts and PDL cells within the alveolar periodontium, where PTH signaling through the cAMP/PKA and PKC pathways modulates the RANKL/OPG ratio in a manner that is dose-dependent and highly context-sensitive [135, 136]. Intermittent, low-amplitude PTH signaling favors bone anabolism by promoting osteoblast differentiation and suppressing osteoblast apoptosis; conversely, sustained or chronically elevated PTH signaling, as occurs in primary and secondary hyperparathyroidism, shifts the RANKL/OPG balance decisively toward osteoclastogenesis, accelerating alveolar bone resorption in a manner that is mechanistically additive to the RANKL upregulation already driven by IL-1β, IL-6, and TNF-α in periodontitis-affected tissues [91, 137]. The convergence of hormonal and inflammatory RANKL induction at the same molecular target, the RANK receptor on osteoclast precursors, means that patients with concurrent hyperparathyroidism and periodontitis face a compounded osteoclastogenic stimulus whose clinical severity may substantially exceed that predicted by either condition alone [138, 139].

Vitamin D, functioning as both a nutrient and a steroid hormone, exerts immunomodulatory, anti-inflammatory, and bone-protective effects in the periodontium that are among the most extensively documented of any endocrine signal in this tissue. The VDR, a nuclear receptor activated by calcitriol, is expressed in gingival keratinocytes, fibroblasts, macrophages, dendritic cells, T lymphocytes, osteoblasts, and PDL cells, making virtually every cell type of the periodontium directly responsive to vitamin D signaling [44]. Upon calcitriol binding, VDR forms a heterodimer with the retinoid X receptor (RXR) and binds to vitamin D response elements (VDREs) in the promoter regions of target genes, driving the transcription of anti-inflammatory mediators and immunomodulatory proteins while suppressing the transcription of pro-inflammatory cytokines. In gingival macrophages and dendritic cells, VDR activation reduces the production of IL-1β, IL-6, IL-12, and TNF-α, promotes the generation of IL-10 and tolerogenic dendritic cell phenotypes, and enhances the synthesis of antimicrobial peptides including cathelicidin (LL-37) and beta-defensin 2, which directly suppress the growth of key periodontal pathogens including *P. gingivalis* and *Treponema denticola* [140]. In osteoblasts and PDL cells, calcitriol signaling promotes OPG synthesis, reduces basal RANKL expression, and enhances the expression of bone matrix proteins including osteocalcin and bone sialoprotein, collectively supporting alveolar bone density and resistance to resorption. The VDR gene was previously identified as a genetic susceptibility locus for periodontitis, with specific VDR polymorphisms including FokI, BsmI, TaqI, and ApaI variants associated with altered disease risk in multiple populations [3], underscoring that the vitamin D-periodontal relationship is not merely a matter of circulating hormone levels but is also modulated by receptor-level genetic variation that determines the cellular responsiveness to calcitriol signaling.

VDR activation by calcitriol has been shown to directly suppress NLRP3 transcription and inhibit caspase-1 activation in macrophages; vitamin D deficiency therefore removes a critical brake on inflammasome activity in periodontal immune cells, contributing to the elevated IL-1β and IL-18 that characterize vitamin D-deficient periodontal inflammation [141].

Vitamin D deficiency, defined as serum 25-hydroxyvitamin D concentrations below 20 ng/mL and highly prevalent in global populations including those at elevated periodontal risk, impairs each of the protective mechanisms described above, generating a state of heightened periodontal inflammatory susceptibility and reduced bone anabolic capacity. In the absence of adequate calcitriol signaling, the antimicrobial peptide production of gingival epithelial cells is diminished, reducing the barrier defense against periodontal pathogens at the dentogingival interface [142, 143]. Macrophage polarization shifts toward a pro-inflammatory M1 phenotype with enhanced NF-κB activity, IL-1β, and IL-6 secretion, amplifying the inflammatory response to subgingival bacteria beyond homeostatic thresholds. The Treg/Th17 balance, which vitamin D normally maintains in part by promoting FoxP3 expression in regulatory T cells and suppressing RORγt-driven Th17 differentiation, shifts toward Th17 dominance, elevating local IL-17 concentrations and thereby increasing neutrophil recruitment, MMP expression, and RANKL-mediated osteoclastogenesis, the precise cascade of events that characterizes destructive periodontitis progression [144].

Epidemiological evidence is consistent with these mechanistic predictions: cross-sectional studies in large population cohorts demonstrate inverse associations between serum vitamin D levels and periodontitis prevalence and severity, and intervention studies with vitamin D supplementation, while methodologically heterogeneous, show trends toward reduced gingival inflammation, lower GCF cytokine concentrations, and modest improvements in clinical attachment in deficient individuals following restoration of adequate vitamin D status [141, 145–147].

Secondary hyperparathyroidism, arising as a compensatory response to chronic vitamin D deficiency or to impaired renal phosphate excretion in chronic kidney disease, deserves particular attention in the context of periodontal-endocrine interactions because it integrates the pathological consequences of both vitamin D insufficiency and PTH excess simultaneously within the same patient. In secondary hyperparathyroidism, chronically reduced calcitriol levels fail to adequately suppress PTH secretion, leading to sustained parathyroid gland hyperplasia and persistent PTH elevation that drives continuous osteoclastic activity throughout the skeleton, including the alveolar bone [49, 148, 149]. The resulting bone metabolic state combines the loss of vitamin D-mediated OPG induction, the loss of calcitriol anti-inflammatory effects in periodontal immune cells, and the gain of PTH-mediated RANKL upregulation in osteoblasts and PDL cells, creating a uniquely hostile molecular environment for alveolar bone maintenance. Secondary hyperparathyroidism was identified as a condition sharing associated genes with periodontitis in the molecular comorbidity network [4], and the shared molecular architecture includes genes involved in bone metabolism, immune regulation, and inflammatory signaling whose co-dysregulation in both conditions compounds the risk of severe alveolar bone loss. Patients with chronic kidney disease, in whom secondary hyperparathyroidism is nearly universal in advanced stages, represent a clinically important population in whom the endocrine-periodontal interaction is particularly severe and in whom vitamin D supplementation and PTH management may have direct periodontal protective benefits beyond their established skeletal and cardiovascular indications [150–152].

The therapeutic potential of targeting the PTH-vitamin D axis for periodontal benefit is beginning to attract systematic investigative attention. Vitamin D supplementation, either alone or in combination with calcium, has been evaluated as an adjunct to conventional periodontal therapy in several randomized controlled trials, with results generally indicating modest but consistent reductions in GCF inflammatory cytokine levels, improved clinical attachment outcomes in vitamin D-deficient subjects, and reduced alveolar bone loss progression in patients with concurrent osteoporosis or metabolic bone disease. The biological plausibility of these benefits is well-supported by the receptor-level mechanisms described above, and the favorable safety profile of vitamin D supplementation at recommended doses makes it an attractive candidate for formal inclusion in periodontal therapeutic protocols for patients with documented deficiency [153].

Analogs of PTH, including teriparatide, which have been approved for the treatment of severe osteoporosis based on their intermittent anabolic bone effects, are being investigated in experimental periodontal models for their potential to promote alveolar bone regeneration through PDL cell stimulation and osteoblast recruitment, with promising results in ligature-induced periodontitis animal models. The clinical translation of PTH analog therapy to periodontal indications remains a future prospect that will require dedicated randomized trials in human subjects, but the molecular rationale is grounded in the well-characterized expression of PTH1R in PDL and alveolar bone cells and the known anabolic effects of intermittent PTH signaling on these receptor-expressing populations. Taken together, the PTH and vitamin D axes represent endocrine regulatory systems whose perturbation contributes meaningfully to periodontal pathogenesis through both direct bone metabolic effects and indirect immunomodulatory mechanisms, and whose restoration to physiological function offers a biologically rational strategy for reducing periodontal inflammatory burden in patients with concurrent mineral metabolism disorders [154–156].

The reciprocal dimension of this relationship, in which chronic periodontal inflammation influences mineral metabolism and the PTH-vitamin D axis, has received comparatively limited attention but carries genuine biological plausibility. The systemic inflammatory state sustained by chronic periodontitis, characterized by persistently elevated IL-1β, IL-6, and TNF-α, can suppress renal 1-alpha-hydroxylase activity, thereby reducing calcitriol production and contributing to functional vitamin D insufficiency even in patients with adequate 25-hydroxyvitamin D substrate levels. Periodontal bacteremia and LPS-driven systemic inflammation may also alter the expression of the calcium-sensing receptor (CaSR) in parathyroid chief cells, potentially modifying the PTH secretory response to calcium signals and subtly disturbing the feedback regulation of the PTH-vitamin D axis over extended periods of chronic oral infection. While the clinical magnitude of these reverse effects remains to be quantified in dedicated studies, they are consistent with the bidirectional model of endocrine-periodontal crosstalk that informs the broader framework of this review, and they suggest that achieving periodontal disease control may contribute to normalizing mineral metabolism parameters in patients with borderline vitamin D status or compensated secondary hyperparathyroidism [157, 158].

## 8 The RANKL/OPG axis as a molecular hub linking endocrine and periodontal pathways

The receptor activator of nuclear factor κB ligand (RANKL) and its decoy receptor osteoprotegerin (OPG) constitute a binary molecular switch whose ratio at the level of the alveolar bone microenvironment determines the net balance between osteoclastic resorption and osteoblastic formation in the periodontium. RANKL, a member of the TNF superfamily expressed on the surface of osteoblasts, PDL cells, activated T lymphocytes, and stromal fibroblasts, binds to its cognate receptor RANK on osteoclast precursors, triggering a signaling cascade through tumor necrosis factor receptor-associated factor 6 (TRAF6), NF-κB, and the nuclear factor of activated T cells 1 (NFATc1) that drives osteoclast differentiation, activation, and survival. OPG, secreted by osteoblasts, PDL fibroblasts, and endothelial cells, competitively inhibits this interaction by acting as a soluble decoy receptor that sequesters RANKL before it can engage RANK, thereby restraining osteoclastogenesis [159, 160].

It is essential to emphasize that while the RANKL/OPG axis provides a particularly tractable molecular framework for understanding how endocrine signals converge on alveolar bone homeostasis, it represents only one component—albeit a central one—of the broader pathophysiology of periodontal disease. Periodontal destruction is a multifactorial process that additionally involves subgingival microbial dysbiosis and the virulence strategies of keystone pathogens such as Porphyromonas gingivalis; epithelial barrier dysfunction and increased mucosal permeability; dysregulated neutrophil recruitment and NET formation; complement pathway activation and opsonin-mediated microbial evasion; macrophage polarization dynamics; the balance between Th1, Th2, Th17, and regulatory T-cell responses; fibroblast-mediated ECM remodeling and MMP activation; and tissue proteolysis by both host-derived and bacterially secreted enzymes. The emphasis on RANKL/OPG in the present review reflects the exceptional density of endocrine hormone inputs to this specific molecular axis and its direct clinical relevance to the alveolar bone loss that defines periodontitis staging and severity; it is not intended to imply that this axis alone explains periodontal pathogenesis or that therapeutic targeting of RANKL/OPG would be sufficient as a standalone intervention [159, 161].

**Figure 2:**
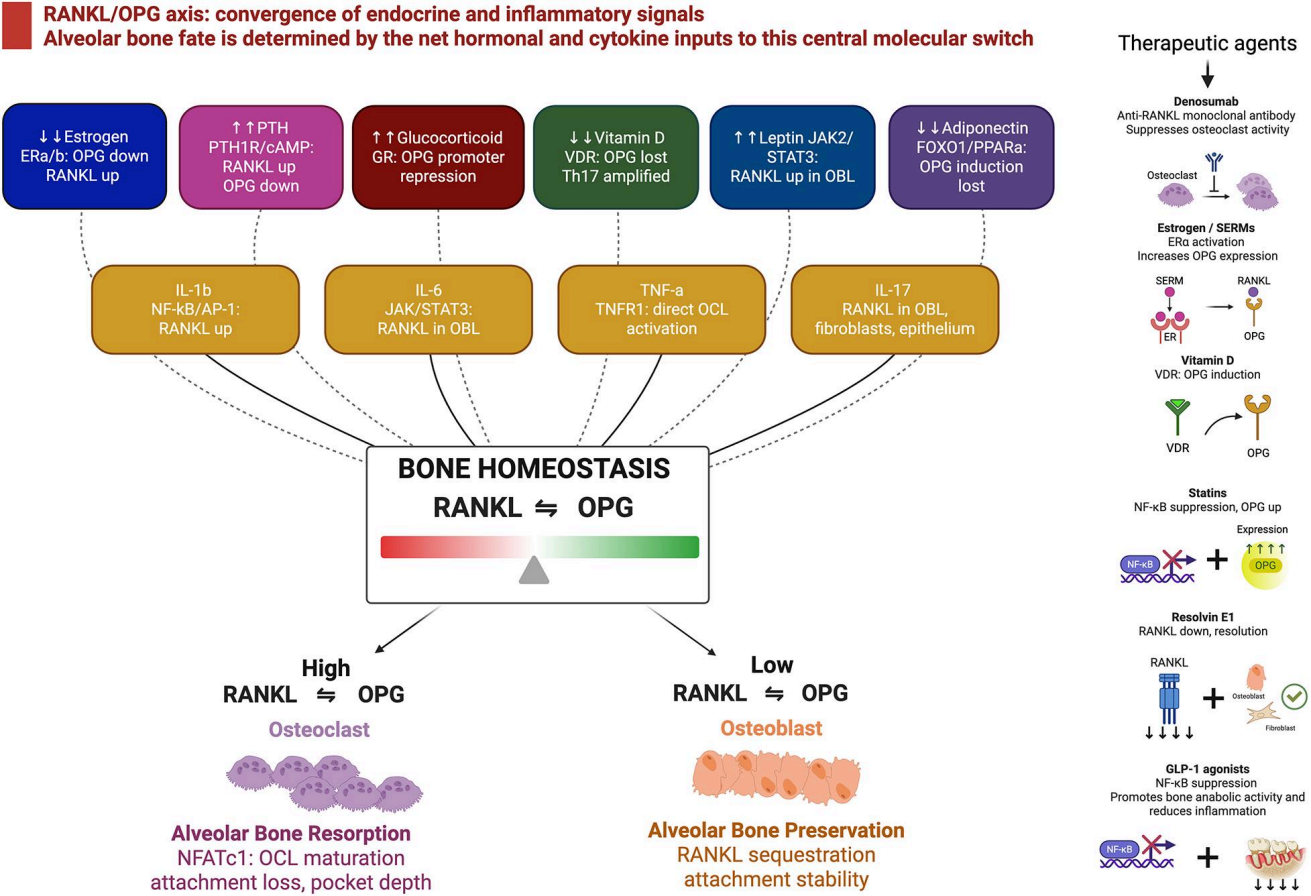
The RANKL/OPG axis as a molecular hub integrating endocrine and inflammatory signals to determine alveolar bone outcomes. Created in https://BioRender.com.

The RANKL/OPG ratio is therefore not a fixed tissue property but a dynamically regulated molecular balance that integrates the full spectrum of anabolic and catabolic signals reaching the alveolar bone, including those originating from both inflammatory mediators and hormonal inputs (See Figure 2). What makes this axis particularly relevant to the present discussion is that virtually every endocrine system reviewed in the preceding sections converges on the RANKL/OPG ratio as a shared downstream effector, such that hormonal dysregulation of multiple origins ultimately translates into periodontal bone vulnerability through this common molecular node. The RANKL/OPG axis thus functions not merely as a bone remodeling regulator but as a molecular integrator of endocrine and inflammatory signals whose collective output determines the pace and extent of alveolar bone destruction in periodontitis [160, 162].

The convergence of hormonal signals on the RANKL/OPG axis is remarkably consistent across endocrine systems despite the diversity of upstream receptor mechanisms involved. Estrogen deficiency reduces OPG secretion by osteoblasts and PDL cells through loss of ERα-mediated transcriptional activation of the OPG gene promoter, simultaneously permitting increased RANKL expression in osteoblasts and activated T cells through derepression of NF-κB and AP-1 target sites; the net result is an elevation in the RANKL/OPG ratio that accelerates alveolar bone resorption in postmenopausal and surgically castrated states [12]. Excess PTH, as occurs in primary and secondary hyperparathyroidism, upregulates RANKL expression in osteoblasts and PDL cells through cAMP/PKA-mediated activation of transcription factors that bind to RANKL gene promoter regions, while simultaneously suppressing OPG production, generating a hormonal RANKL/OPG imbalance that is additive to the inflammatory imbalance already driven by periodontal cytokines [159, 162].

Glucocorticoid excess suppresses OPG synthesis in osteoblasts through GR-mediated repression of the OPG promoter while upregulating RANKL expression in both osteoblasts and T cells, producing a glucocorticoid-specific pattern of RANKL/OPG dysregulation that explains the well-documented association between corticosteroid therapy and accelerated alveolar bone loss. Vitamin D deficiency, by reducing calcitriol-driven OPG induction in osteoblasts and failing to suppress Th17-derived IL-17 that further upregulates RANKL in stromal cells, also shifts the RANKL/OPG balance toward resorption through a mechanism that involves both direct transcriptional effects and indirect immunological amplification. Leptin, signaling through JAK2/STAT3 in osteoblasts, upregulates RANKL expression while having neutral or modestly suppressive effects on OPG, whereas adiponectin, through FOXO1 and PPARα activation, promotes OPG synthesis and inhibits osteoclast precursor differentiation, establishing the adipokine balance as an additional hormonal determinant of the RANKL/OPG ratio in periodontal bone [163–165].

The inflammatory cytokines that are overproduced in periodontitis converge on the same RANKL/OPG target through distinct but mechanistically complementary pathways that synergize with the hormonal inputs described above. IL-1β, acting through IL-1R1 on osteoblasts and PDL cells, activates NF-κB and AP-1 to dramatically upregulate RANKL transcription while suppressing OPG expression, producing one of the most potent shifts in RANKL/OPG ratio of any single mediator in the periodontal context. IL-6, signaling through its receptor and the gp130 co-receptor via JAK/STAT3, similarly upregulates RANKL in osteoblasts and PDL fibroblasts while promoting the differentiation of osteoclast precursors independently of RANKL through a soluble RANKL-like mechanism [162].

TNF-α, whose gene is the most highly connected hub in the periodontitis molecular comorbidity network with a connectivity degree of 24 disease associations [4], synergizes with IL-1β to amplify RANKL expression and directly activates osteoclast precursors through TNFR1 in a RANKL-independent manner, further compounding the osteoclastogenic stimulus. IL-17, produced by Th17 cells whose differentiation is promoted by the IL-6 and IL-23 that are abundantly expressed in periodontal lesions, upregulates RANKL in osteoblasts, fibroblasts, and epithelial cells through multiple downstream signaling pathways and simultaneously induces the production of additional IL-1β and TNF-α, creating a feed-forward inflammatory loop whose terminus is consistently osteoclast activation and alveolar bone resorption [144]. The implication of this convergence is that the inflammatory and hormonal RANKL/OPG dysregulations that occur simultaneously in patients with periodontitis and concurrent endocrine disease do not simply add arithmetically but interact multiplicatively, as each hormonal shift lowers the threshold at which inflammatory cytokines can tip the RANKL/OPG balance beyond the point of effective compensatory OPG secretion [162].

The molecular architecture of the RANKL/OPG system also incorporates a number of regulatory inputs from the extracellular environment that further link endocrine signals to periodontal bone vulnerability. The Wnt/β-catenin signaling pathway, which promotes OPG expression in osteoblasts and PDL cells while suppressing RANKL, is inhibited by the endogenous Wnt antagonist Dickkopf-1 (DKK1), whose expression is upregulated by pro-inflammatory cytokines including IL-1β, TNF-α, and IL-6 in periodontal tissues [166]. Recent evidence from inflammatory arthritis models demonstrates that DKK1 levels positively correlate with IL-6 concentrations and disease activity, and that targeting the Wnt pathway represents a convergence point between inflammatory cytokine networks and bone metabolic dysregulation, a mechanism directly applicable to periodontitis given the shared cytokine profiles of these two conditions [4].

Sclerostin, another Wnt antagonist produced by osteocytes in response to mechanical unloading and inflammatory cytokines, similarly suppresses OPG production by osteoblasts and has been detected at elevated levels in the GCF of patients with severe periodontitis, suggesting that the local osteocyte response to periodontal inflammation further amplifies the osteoclastogenic shift through Wnt pathway inhibition. AGEs, generated in abundance in the hyperglycemic periodontal microenvironment of diabetic patients, activate RAGE on osteoblasts to suppress Wnt/β-catenin signaling and reduce OPG synthesis, linking the insulin resistance axis directly to RANKL/OPG dysregulation through a mechanism that is independent of cytokine-mediated RANKL upregulation. These regulatory layers transform the RANKL/OPG axis from a simple binary switch into a highly sensitive integrator of endocrine, metabolic, inflammatory, and mechanical signals whose net output reflects the aggregate hormonal and immunological status of the organism at any given time [167, 168].

The clinical implications of the RANKL/OPG axis as a molecular hub are substantial for both the diagnosis and therapeutic management of patients with concurrent periodontal disease and endocrine disorders (Some common therapeutic agents and their targets are displayed in Figure 2). Measurement of OPG and soluble RANKL concentrations in GCF and serum has been proposed as a composite diagnostic approach for assessing the severity of alveolar bone resorptive activity in periodontitis, and the RANKL/OPG ratio in GCF correlates with radiographic bone loss parameters more strongly than either molecule measured in isolation. The potential use of this ratio as a molecular biomarker of periodontal-endocrine interaction, reflecting the combined contribution of hormonal dysregulation and local inflammatory activation to bone resorptive risk, represents a clinically actionable application of the mechanistic framework developed in this review [159]. From a therapeutic standpoint, pharmacological agents that modify RANKL/OPG signaling include the monoclonal antibody denosumab, which binds and neutralizes RANKL and is currently approved for osteoporosis and bone metastasis management. In preclinical models, subcutaneous denosumab administration has been shown to significantly reduce alveolar bone loss and osteoclast numbers in ligature-induced experimental periodontitis in rodents [169] . Human evidence remains limited to case reports and secondary observations in patients receiving denosumab for oncological or osteoporosis indications, and no dedicated randomized controlled trial has evaluated denosumab as a periodontal adjunct; its potential in this context therefore remains exploratory and requires formal clinical investigation.

Host modulation therapies that enhance OPG production or suppress RANKL expression in periodontal cells, including statins, bisphosphonates, and resolvin E1, have similarly shown promise in experimental models and align mechanistically with the RANKL/OPG-centered framework presented here [170].

The recognition that multiple endocrine systems converge on this single molecular axis in the periodontium implies that integrated management of hormonal dysregulation, whether through endocrine therapy, metabolic optimization, or nutritional supplementation, may reduce RANKL/OPG-mediated alveolar bone loss through additive or synergistic mechanisms that conventional periodontal treatment alone cannot adequately address [159].

The position of the RANKL/OPG axis as a molecular convergence point also illuminates why periodontitis so consistently co-occurs with the endocrine conditions reviewed throughout this article, and why its severity so often correlates with the degree of hormonal dysregulation rather than simply with the magnitude of the local microbial challenge.

In patients with postmenopausal estrogen deficiency, insulin resistance, secondary hyperparathyroidism, glucocorticoid excess, thyroid dysfunction, or adipokine imbalance, the RANKL/OPG ratio in alveolar bone is pre-shifted toward osteoclastic activity before any inflammatory stimulus from subgingival biofilms is applied. When periodontal inflammation is superimposed on this hormonally primed environment, the cytokine-driven RANKL upregulation encounters a tissue whose compensatory OPG-secreting capacity is already compromised, generating a degree of alveolar bone loss that is disproportionate to the apparent severity of the local infection. This concept of hormonal priming of the RANKL/OPG axis represents one of the most mechanistically coherent explanations for the clinical observation that patients with endocrine disorders exhibit more severe and more rapidly progressive periodontitis than their microbiologically comparable euthyroid, insulin-sensitive, or premenopausal counterparts, and it provides a unifying molecular rationale for the integrated endocrine-periodontal management strategies discussed in the concluding sections of this review [8].

## 9 Molecular network perspective: shared pathways between endocrine dysregulation and periodontitis

The preceding sections have examined individual endocrine axes in their relationship to periodontal pathogenesis, tracing specific receptor-mediated mechanisms through which hormonal imbalance modifies the inflammatory, immune, and bone metabolic environment of the periodontium. While this axis-by-axis approach is analytically necessary for mechanistic clarity, it risks obscuring a fundamental property of the endocrine-periodontal relationship: that the molecular interactions involved are not parallel and independent but form an integrated, highly interconnected network in which dysregulation of one hormonal axis predictably alters the signaling landscape of others, and in which the inflammatory consequences of periodontal disease reverberate across multiple endocrine systems simultaneously. A network-based perspective, grounded in the systematic analysis of gene-disease associations and molecular pathway co-enrichment, provides an indispensable complementary lens through which the systemic nature of this relationship becomes fully visible. A comprehensive molecular comorbidity analysis of periodontitis, employing curated gene-disease association mining across the OMIM, PubMed, and DisGeNET databases, revealed that the periodontitis diseasome encompasses 2,219 curated gene-disease interactions involving 80 distinct disease conditions, organized in a highly interconnected network whose core is dominated by inflammatory, metabolic, and endocrine signaling molecules [4].

Among the 80 conditions sharing molecular players with periodontitis, a substantial subset belongs to the endocrine and metabolic disease categories, including obesity, non-insulin-dependent diabetes mellitus, polycystic ovary syndrome, adrenal gland hypofunction, secondary hyperparathyroidism, and diabetic retinopathy and neuropathy, confirming that the endocrine-periodontal relationship identified through clinical and experimental research has a robust molecular foundation detectable through unbiased database interrogation. The hub genes of the periodontitis molecular comorbidity network illuminate the specific molecular nodes through which endocrine and periodontal pathologies are most deeply interconnected. Tumor necrosis factor (TNF), the most highly connected hub gene with a degree of 24 disease associations spanning rheumatoid arthritis, ulcerative colitis, diabetes mellitus, and myocardial infarction, encodes a cytokine that simultaneously serves as the principal driver of periodontal RANKL upregulation, a mediator of insulin resistance through IRS-1 serine phosphorylation, a suppressor of adiponectin secretion by adipocytes, and a promoter of HPA axis activation through hypothalamic CRH stimulation [4].

Interleukin-6 (IL6), the second most connected hub with 23 disease associations including Crohn disease, breast adenocarcinoma, and atherosclerosis, similarly bridges periodontal and endocrine pathology through its dual roles as an inducer of hepatic acute-phase proteins that impair insulin signaling and as a promoter of Th17 cell differentiation that amplifies RANKL-mediated osteoclastogenesis in both articular and alveolar bone [4, 144].

Prostaglandin-endoperoxide synthase 2 (PTGS2), encoding cyclooxygenase-2, connects periodontitis to depressive disorder, cerebrovascular accident, and coronary artery disease through prostaglandin E2-mediated vascular and neuroendocrine effects that include modulation of HPA axis reactivity and alteration of hypothalamic prostaglandin signaling involved in fever and stress responses. The STAT3, a convergence node for both cytokine receptor and hormone receptor signaling, is activated downstream of LEP, IL-6, GH, prolactin, and insulin pathways, and its connectivity across 11 disease conditions in the periodontitis diseasome reflects its position as a molecular crossroads between endocrine and inflammatory signaling that is simultaneously relevant to periodontal pathogenesis, adipokine dysregulation, and oncogenic processes [4, 171].

The LEP gene and NOS3 deserve particular attention as hub genes whose connectivity pattern most directly maps the endocrine-periodontal interface within the diseasome network. The LEP gene, with connectivity spanning obesity, type 2 diabetes, depressive disorder, and non-alcoholic fatty liver disease, encodes the principal adipose-derived hormone whose pro-inflammatory JAK2/STAT3 signaling in periodontal macrophages and fibroblasts has been described in detail in the preceding sections. Its position as a hub gene in the periodontitis molecular network means that the metabolic conditions in which LEP is elevated or in which LEP receptor signaling is dysregulated, principally obesity and the leptin resistance that accompanies it, are predicted by network topology to share not merely correlative but mechanistically grounded molecular connections with periodontitis.

NOS3, encoding endothelial nitric oxide synthase, connects periodontitis to Alzheimer’s disease, diabetes mellitus, coronary artery disease, and breast neoplasms through nitric oxide-mediated vascular and immune regulatory mechanisms [4, 172]. In the periodontal context, NOS3-derived nitric oxide participates in the regulation of gingival vascular tone, modulates macrophage bactericidal activity against periodontal pathogens, and interacts with estrogen receptor signaling to mediate the vasodilatory and anti-inflammatory effects of estrogen in vascular endothelium, creating a molecular bridge between the estrogenic and vascular endocrine axes that converges on the periodontal microvasculature. The reduction of NOS3 activity observed in states of insulin resistance, estrogen deficiency, and hypercortisolism thus represents a shared mechanism through which multiple endocrine perturbations simultaneously impair vascular and immune function in periodontal tissues. Functional enrichment analysis of the genes comprising the periodontitis diseasome reveals that the shared molecular pathways most significantly over-represented across periodontitis and its comorbid conditions are precisely those that mediate endocrine-immune crosstalk at the molecular level [4].

The PI3K/Akt signaling pathway, enriched with an adjusted p-value of $2.5\times 10^{-25}$, is activated downstream of insulin, IGF-1, LEP, and multiple growth factor receptors, and mediates the anabolic, anti-apoptotic, and anti-inflammatory effects of these hormones in periodontal cells; its dysregulation in insulin resistance and metabolic syndrome directly impairs the trophic and cytoprotective signaling that normally maintains periodontal tissue homeostasis. The AGE-RAGE signaling pathway in diabetic complications, enriched with an adjusted p-value of $5.8\times 10^{-23}$, represents the molecular mechanism through which chronic hyperglycemia converts systemic metabolic dysregulation into amplified local periodontal NF-κB activation, as described in the insulin resistance section [172]. The JAK-STAT signaling pathway, enriched at $9.7\times 10^{-16}$, is the principal transducer of LEP, IL-6, interferon, and prolactin signals in periodontal immune cells, and its constitutive activation in states of hormonal and inflammatory excess drives the chronic pro-inflammatory gene expression programs that sustain periodontitis. The Th17 cell differentiation pathway, enriched at $1.1\times 10^{-14}$, is promoted by IL-6 and IL-23 in contexts of both periodontal infection and endocrine dysregulation including PCOS and estrogen deficiency, linking adaptive immune polarization to hormonal status through shared cytokine signals [171]. The Hypoxia-inducible factor 1 (HIF-1) signaling pathway, enriched at $9.7\times 10^{-15}$, is activated by inflammatory cytokines, and insulin resistance to promote angiogenesis, glycolytic metabolism, and inflammatory gene expression in periodontal tissues, and its co-enrichment with vascular endothelial growth factor A (VEGFA), another hub gene in the periodontitis diseasome, connects the vascular endocrine axis to the molecular pathogenesis of periodontal disease through hypoxia-driven inflammation [4].

The regulatory non-coding RNA landscape of the periodontitis diseasome adds a further dimension of molecular connectivity between endocrine and periodontal pathological processes. Analysis of the periodontitis comorbidity network revealed 38 microRNAs capable of regulating large numbers of the hub genes identified in the diseasome, including miR-146a, which targets key components of the NF-κB signaling pathway including TRAF6 and interleukin 1 receptor-associated kinases (IRAK1), limiting the production of IL-1β and TNF-α in periodontal macrophages while also modulating insulin receptor signaling and adipokine-driven inflammatory responses [4]. MiR-21, upregulated in periodontitis tissues, suppresses PTEN and thereby promotes PI3K/Akt-driven inflammatory macrophage activation while simultaneously impairing the insulin sensitizing functions of this pathway in metabolically active tissues, representing a microRNA-level mechanism that simultaneously amplifies periodontal inflammation and worsens systemic insulin resistance [173, 174]. MiR-155, induced by LPS and pro-inflammatory cytokines in periodontal macrophages, targets the glucocorticoid receptor itself, reducing GR expression and thereby impairing the anti-inflammatory GR-mediated transrepression of NF-κB in periodontal cells, which represents a molecular mechanism through which established periodontal inflammation can render itself progressively more resistant to both endogenous cortisol-mediated resolution and exogenous corticosteroid therapy. The hypermethylation of the IL10 gene promoter in periodontal tissues, which reduces the expression of this central anti-inflammatory cytokine and was previously identified as an epigenetic mechanism contributing to periodontitis pathogenesis, is promoted by the same inflammatory cytokine milieu that characterizes insulin resistance, hypercortisolism, and estrogen deficiency, suggesting that epigenetic silencing of anti-inflammatory genes represents a molecular mechanism through which multiple endocrine perturbations converge to impair periodontal resolution capacity [175].

The network perspective ultimately reveals that the endocrine-periodontal relationship is not a collection of pairwise associations between isolated hormonal systems and a single oral inflammatory condition, but rather an expression of deeply shared molecular architecture in which inflammatory, metabolic, and hormonal signaling networks are so extensively interconnected that dysregulation at any node propagates consequences throughout the system. The periodontitis diseasome, with its 506 health conditions reported as significantly enriched in the gene sets of periodontitis-associated diseases [4], can be understood as a molecular map of the systemic consequences of this interconnectedness, in which endocrine disorders occupy a central and structurally important position. This systems-level understanding carries direct implications for clinical management: it suggests that interventions targeting a single molecular node, whether antimicrobial, anti-inflammatory, or hormonal, are unlikely to fully interrupt the self-reinforcing network dynamics that sustain both periodontitis and its endocrine comorbidities. Instead, therapeutic strategies that simultaneously address multiple nodes of the shared molecular network, combining optimized periodontal treatment with endocrine management, metabolic optimization, and targeted modulation of shared hub gene products such as TNF, IL-6, and STAT3, are more likely to produce durable improvements in both oral and systemic health outcomes. The molecular network perspective thus provides not only a richer understanding of disease pathogenesis but also a principled framework for designing the integrated, interdisciplinary therapeutic approaches that the complexity of the endocrine-periodontal relationship demands [176, 177].

## 10 Bidirectionality: how periodontal inflammation disrupts endocrine function

**Figure 3:**
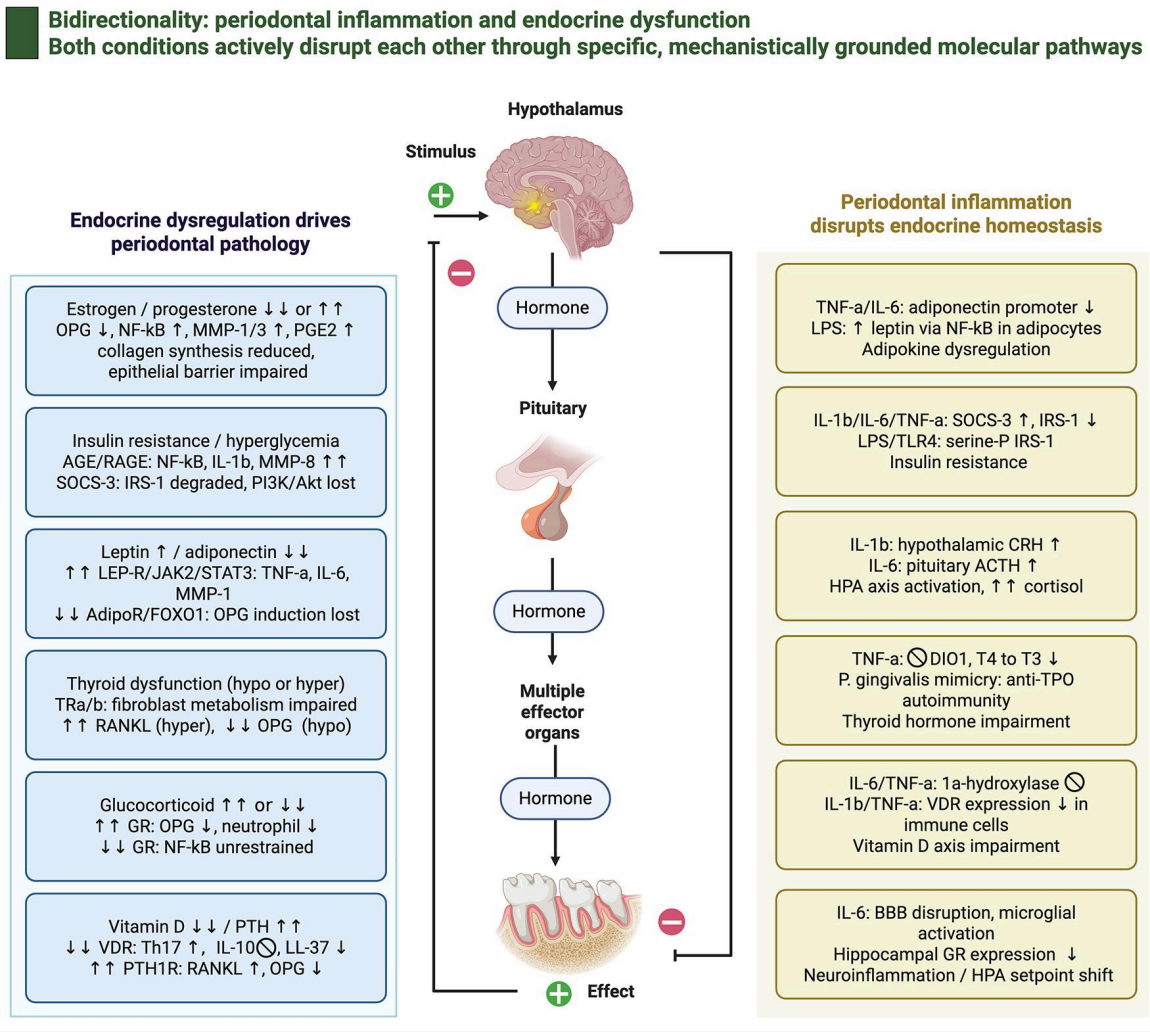
Bidirectionality between periodontal inflammation and endocrine dysfunction: molecular mechanisms of reciprocal disruptions. Created in https://BioRender.com.

The preceding sections have primarily examined how hormonal imbalance modifies periodontal susceptibility, progression, and tissue-level molecular responses. However, the relationship between periodontitis and endocrine dysfunction is not unidirectional, and a mechanistically complete account of this interaction must equally address how chronic periodontal inflammation disrupts endocrine function at the systemic level [51]. This reverse directionality is not a minor or secondary consideration; it is a central feature of the pathological coupling between oral and systemic health that transforms periodontitis from a locally confined inflammatory disease into a systemic modifier of hormonal homeostasis (Figure 3). The biological plausibility of this reverse causation rests on several well-established facts: the ulcerated pocket epithelium of periodontitis patients provides a substantial surface area for the direct translocation of bacterial products and inflammatory mediators into the systemic circulation; bacteremic episodes associated with periodontal manipulation and even routine activities such as chewing and toothbrushing introduce periodontal pathogens and their endotoxins into the bloodstream multiple times daily; and the chronically elevated concentrations of IL-1β, IL-6, TNF-α, and prostaglandins that characterize active periodontitis produce systemic effects on endocrine organs, receptor expression, and hormonal signaling pathways that are measurable in both experimental models and clinical studies [3, 4].

Understanding these reverse mechanisms is essential not only for scientific completeness but for appreciating the clinical urgency of periodontal disease control in patients with concurrent endocrine disorders. The disruption of insulin signaling and glycemic homeostasis by periodontal inflammation represents the most extensively documented example of reverse endocrine-periodontal causation, and the one for which the molecular mechanisms are most precisely characterized. Systemically disseminated IL-1β, IL-6, and TNF-α from periodontal tissues activate SOCS-3 expression in hepatocytes, skeletal muscle cells, and adipocytes, directly impairing IRS-1 phosphorylation and uncoupling the insulin receptor from its downstream PI3K/Akt effector cascade in peripheral tissues far removed from the oral cavity [67, 178]. Simultaneously, periodontal LPS, predominantly derived from the outer membrane of gram-negative anaerobes including *P. gingivalis*, activates TLR4 on adipocytes and skeletal muscle cells to induce serine phosphorylation of IRS-1, a modification that prevents its productive interaction with the activated insulin receptor and thereby reduces glucose uptake in peripheral tissues. The net result of these two parallel mechanisms is a state of systemic insulin resistance whose magnitude correlates with the severity and extent of periodontal inflammation, explaining the consistent finding in large epidemiological studies that poorly controlled periodontitis is associated with elevated HbA1c levels, impaired fasting glucose, and increased insulin requirements even in patients without a prior diagnosis of diabetes [179].

Crucially, successful non-surgical periodontal therapy is associated with modest but consistent reductions in HbA1c, circulating IL-6, CRP, and TNF-α, and partial restoration of insulin sensitivity, providing interventional evidence that confirms the causal contribution of periodontal inflammation to systemic insulin resistance and supporting the concept that periodontal treatment constitutes a meaningful adjunct to glycemic management in patients with type 2 diabetes [3, 179].

The impact of periodontal inflammation on adipokine profiles represents a less intuitive but mechanistically important form of reverse endocrine disruption. Chronically elevated periodontal-derived TNF-α and IL-6 suppress adiponectin gene expression in adipocytes through NF-κB-mediated transcriptional repression of the adiponectin promoter, contributing to the low circulating adiponectin levels observed in patients with severe periodontitis even in the absence of obesity or metabolic syndrome [71]. This inflammation-driven suppression of adiponectin has consequences that extend beyond the periodontium itself, as reduced systemic adiponectin further impairs insulin sensitivity in peripheral tissues, reduces anti-inflammatory tone in vascular endothelium, and diminishes the protective effects of adiponectin on osteoclastogenesis at skeletal sites throughout the body, illustrating how periodontal inflammation can propagate metabolic and endocrine consequences through the adipokine network [180].

Conversely, periodontal LPS and inflammatory cytokines stimulate LEP secretion by adipocytes through NF-κB-dependent mechanisms, elevating circulating LEP concentrations and promoting the JAK2/STAT3-driven pro-inflammatory signaling that this adipokine exerts on immune cells and fibroblasts throughout the organism. The periodontal-driven shift in the leptin-to-adiponectin ratio, toward a more pro-inflammatory and insulin-resistant hormonal profile, exemplifies how localized oral infection can reshape the systemic adipokine milieu in ways that promote metabolic deterioration and amplify the inflammatory burden of concurrent endocrine conditions. These bidirectional adipokine dysregulations are consistent with the hub gene connectivity of LEP in the periodontitis diseasome, which links periodontitis molecularly to obesity, type 2 diabetes, and non-alcoholic fatty liver disease through shared inflammatory and adipokine signaling circuits [4, 171].

Thyroid hormone metabolism is another endocrine system demonstrably affected by the systemic consequences of chronic periodontal inflammation. Periodontal-derived IL-1β and IL-6 can suppress the expression of TSHR on thyroid follicular cells and reduce the activity of TPO, the enzyme responsible for thyroid hormone synthesis, through mechanisms that involve both direct cytokine-mediated transcriptional repression and oxidative inactivation of the enzyme by ROS generated in the context of systemic inflammatory activation. TNF-α, at concentrations achievable in the systemic circulation of patients with severe generalized periodontitis, inhibits the conversion of T4 to the biologically active T3 by suppressing the activity of type 1 deiodinase (DIO1) in hepatocytes and peripheral tissues, contributing to a pattern of low T3 syndrome or non-thyroidal illness that has been documented in patients with severe chronic inflammatory conditions [181].

Furthermore, the molecular mimicry potential of *P. gingivalis* protein epitopes, which share structural homology with thyroid antigens including thyroid peroxidase and thyroglobulin, raises the possibility that periodontal bacteremia in genetically susceptible individuals may trigger or exacerbate autoimmune thyroid disease through cross-reactive immune responses, a mechanism analogous to the citrullination-based molecular mimicry proposed for the periodontitis-rheumatoid arthritis relationship [182, 183]. While direct causal evidence for periodontal bacteremia-driven thyroid autoimmunity in humans remains limited and requires prospective investigation, bidirectional Mendelian randomization studies provide genetic-level support for a causal relationship between periodontal disease and thyroid dysfunction that cannot be attributed solely to shared confounders [3].

The HPA axis, whose role as a modulator of periodontal susceptibility was examined in Section 6, is equally subject to dysregulation by the systemic inflammatory consequences of periodontal disease, completing the bidirectional circuit between these two systems. Circulating IL-1β, IL-6, and TNF-α are well-established activators of the HPA axis at multiple levels: IL-1β stimulates CRH secretion from hypothalamic paraventricular neurons, IL-6 directly stimulates ACTH release from anterior pituitary corticotrophs, and TNF-α enhances the sensitivity of the adrenal cortex to ACTH stimulation, collectively driving cortisol hypersecretion in proportion to the severity of the systemic inflammatory load. In patients with chronic generalized periodontitis, this inflammation-driven HPA activation generates a low-grade but persistent elevation in baseline cortisol that, over time, may contribute to the glucocorticoid receptor resistance patterns and blunted cortisol awakening responses observed in individuals with chronic inflammatory diseases and depression [184].

The neuroinflammatory consequences of periodontal bacteremia and cytokine spillover, including the IL-6-mediated disruption of the blood-brain barrier previously documented in experimental models [3], may additionally alter hippocampal glucocorticoid receptor expression and impair the negative feedback regulation of the HPA axis, thereby modifying the cortisol set-point in ways that further compromise immune and metabolic homeostasis [184]. These neuroendocrine effects of periodontal inflammation are consistent with the molecular connectivity between periodontitis, depressive disorder, and Alzheimer’s disease documented in the diseasome network [4], and they suggest that the psychiatric and neurological comorbidities of periodontitis are partly mediated through HPA axis dysregulation driven by the systemic inflammatory consequences of chronic oral infection.

The vitamin D axis is subject to bidirectional disruption by periodontal inflammation through mechanisms that operate at the level of both vitamin D metabolism and receptor expression [44]. Systemically elevated IL-6 and TNF-α, as occur in severe generalized periodontitis, suppress renal 1-alpha-hydroxylase activity, reducing the conversion of 25-hydroxyvitamin D to calcitriol and thereby generating functional vitamin D insufficiency even in patients with adequate sun exposure and dietary vitamin D intake. This inflammation-driven impairment of vitamin D activation creates a state of relative calcitriol deficiency that diminishes the antimicrobial peptide production, the anti-inflammatory macrophage modulation, and the OPG-inducing effects of vitamin D signaling in periodontal tissues, effectively lowering the inflammatory threshold of the periodontium and creating a molecular environment more permissive of progressive disease. Concurrently, pro-inflammatory cytokines including IL-1β and TNF-α downregulate VDR expression in periodontal macrophages and fibroblasts, reducing cellular responsiveness to calcitriol even when circulating levels are adequate, a form of endocrine resistance at the receptor level that further impairs the protective capacity of vitamin D signaling in inflamed periodontal tissues [44]. The bidirectional disruption of the vitamin D axis by periodontal inflammation thus operates through both reduced hormone production and reduced receptor responsiveness, and it implies that optimizing vitamin D status in periodontitis patients may require higher supplementation targets than those recommended for the general population, a clinical consideration that warrants prospective evaluation in trials designed to assess the interaction between vitamin D supplementation dose, baseline inflammatory status, and periodontal treatment outcomes.

The recognition of bidirectionality in the endocrine-periodontal relationship has important and immediate implications for clinical practice that extend beyond the academic characterization of disease mechanisms. If periodontal inflammation actively disrupts insulin signaling, suppresses adiponectin, activates the HPA axis, impairs thyroid hormone metabolism, and reduces functional vitamin D availability, then untreated or poorly controlled periodontitis represents an active and ongoing source of endocrine disruption that undermines the effectiveness of pharmacological and lifestyle interventions targeting these same endocrine systems [185]. Conversely, successful periodontal therapy, by reducing the systemic inflammatory load generated by the periodontal lesion, may improve insulin sensitivity, restore adipokine balance, normalize HPA reactivity, and support more effective vitamin D signaling simultaneously, producing endocrine benefits that are not captured by oral health outcome measures alone. The emerging evidence from Mendelian randomization studies, which provides causal rather than merely associative support for several of these relationships, strengthens the case for treating periodontal disease as a modifiable endocrine risk factor in addition to its well-established role as a systemic inflammatory burden. This bidirectional understanding ultimately demands a fundamental reorientation of how clinicians in both periodontics and endocrinology conceptualize the boundaries of their respective disciplines, recognizing that the oral cavity and the endocrine system are coupled components of an integrated physiological network whose mutual influences cannot be effectively managed in isolation [186, 187].

## 11 Clinical and therapeutic implications

The mechanistic framework developed throughout this review carries substantial and immediate implications for clinical practice in both periodontology and endocrinology, implications that extend well beyond the academic characterization of shared molecular pathways. If endocrine dysregulation and periodontal inflammation are bidirectionally coupled through the molecular mechanisms described in the preceding sections, then the clinical management of patients in whom both conditions coexist requires a fundamentally different approach from that applied to patients with either condition in isolation. The conventional model, in which the periodontist manages oral inflammation independently of systemic endocrine status and the endocrinologist manages hormonal dysregulation without systematic consideration of oral health, is mechanistically insufficient and clinically suboptimal for a growing population of patients whose periodontal disease severity is shaped by hormonal factors and whose endocrine management is complicated by the systemic inflammatory burden of uncontrolled oral infection [188–190].

The integrated clinical model that the evidence demands is one in which periodontal assessment is incorporated into the routine evaluation of patients with endocrine disorders, endocrine status is systematically characterized in patients presenting with severe, rapidly progressive, or treatment-refractory periodontitis, and therapeutic decisions in both specialties are made with explicit awareness of the bidirectional molecular interactions that link them. Realizing this model in practice requires attention to several specific dimensions of diagnosis, biomarker utilization, therapeutic strategy, and interdisciplinary collaboration that are outlined in the following paragraphs. The diagnostic dimension of the endocrine-periodontal relationship begins with the recognition that endocrine status is a clinically meaningful modifier of periodontal disease risk that should be systematically recorded and considered in periodontal risk stratification. Current periodontal risk assessment tools incorporate factors including smoking status, diabetes, genetic susceptibility, previous disease history, and plaque control; the evidence reviewed here supports (see Table 1) the formal inclusion of additional endocrine variables including menopausal status and estrogen replacement therapy, thyroid function, corticosteroid use and adrenal reserve, vitamin D status, adipokine profiles, and the presence of conditions associated with hormonal dysregulation such as PCOS, obesity, and metabolic syndrome [188, 189].

**Table 1:** Summary of evidence levels supporting major endocrine-periodontal associations.

Endocrine condition	Direction of association	Highest level of available evidence	Key caveats
Type 2 diabetes/insulin resistance	Bidirectional	RCT/systematic meta-analysis	Causality partially confirmed by Mendelian randomization; HbA1c improvements after periodontal therapy are modest and heterogeneous
Postmenopausal estrogen deficiency	Endocrine → periodontal	Observational cohort, some RCT evidence for HRT	HRT benefits must be weighed against systemic risks; effect sizes vary across populations
Adverse pregnancy outcomes	Bidirectional (periodontal → obstetric)	Systematic review with inconsistent RCT results	Causality not established; considerable heterogeneity across meta-analyses
Thyroid dysfunction	Bidirectional	Cohort studies, Mendelian randomization	MR studies limited by pleiotropic SNP effects; direct mechanistic evidence mainly experimental
Glucocorticoid excess/HPA dysregulation	Endocrine → periodontal	Observational, experimental	Clinical data largely from cross-sectional studies; few interventional studies available
Vitamin D deficiency	Bidirectional	Cross-sectional epidemiology, some RCT evidence	Supplementation RCTs show heterogeneous outcomes; optimal target levels for periodontal benefit not established
GLP-1 receptor agonists (therapeutic)	Potentially periodontal-protective	Animal models, *in vitro*, observational trends	No dedicated RCT with periodontal primary endpoints; direct human evidence lacking
PTH excess/secondary hyperparathyroidism	Endocrine → periodontal	Observational, mechanistic	Clinical periodontal studies in hyperparathyroidism populations are limited
PCOS/androgen excess	Bidirectional	Observational, case-control	Confounding by insulin resistance difficult to fully separate from androgen effects

The practical implementation of this expanded risk assessment does not necessarily require periodontal clinicians to conduct endocrine evaluations independently, but it does require that they systematically collect relevant medical history, recognize the periodontal significance of endocrine diagnoses communicated by patients or referring physicians, and establish referral pathways to endocrinology when periodontal presentation raises suspicion of undiagnosed or inadequately managed hormonal dysfunction. Conversely, endocrinologists managing patients with diabetes, thyroid disease, adrenal disorders, PCOS, or postmenopausal bone loss should incorporate periodic referral to periodontal evaluation into their standard of care protocols, recognizing that uncontrolled periodontitis in these patients represents an active source of systemic inflammatory disruption that may undermine glycemic control, bone metabolic management, and the effectiveness of hormonal therapies [189].

Molecular and salivary biomarkers offer a promising avenue for translating the mechanistic understanding of endocrine-periodontal interactions into clinically actionable diagnostic tools. The RANKL/OPG ratio in GCF, which integrates the contributions of both hormonal dysregulation and local inflammatory activation to alveolar bone resorptive risk, represents a composite molecular biomarker of particular relevance in patients with concurrent endocrine disease, as it captures the compounded osteoclastogenic stimulus that arises when hormonal priming of the RANKL/OPG axis coincides with cytokine-driven RANKL upregulation from periodontal inflammation [159]. Salivary and GCF concentrations of IL-1β and IL-6, already proposed as diagnostic biomarkers for periodontitis staging, carry additional clinical information in the endocrine context: in patients with insulin resistance, elevated GCF IL-6 reflects both local periodontal inflammation and the systemic low-grade inflammatory state driven by adipose tissue dysfunction, such that its measurement provides simultaneous information about oral disease activity and systemic metabolic inflammatory burden [161]. Adipokine profiling in saliva and serum, including leptin, adiponectin, visfatin, and resistin, may provide complementary diagnostic information about the hormonal-inflammatory status of the periodontium and its systemic context, particularly in patients with obesity, metabolic syndrome, or PCOS in whom adipokine dysregulation is a central pathological feature.

As point-of-care biosensor technologies and multiplex salivary assay platforms become more accessible and clinically deployable, the integration of these molecular biomarkers into chairside periodontal diagnostic workflows becomes increasingly feasible, and the endocrine context provides a particularly compelling indication for their use given the well-characterized hormonal determinants of the molecular signals they measure [191–193].

From a therapeutic standpoint, the endocrine-periodontal relationship provides both a rationale for optimizing hormonal management as an adjunct to conventional periodontal therapy and a justification for incorporating periodontal treatment into the broader therapeutic strategy for endocrine conditions [61]. Conventional non-surgical periodontal therapy, comprising supra- and subgingival debridement with or without adjunctive antimicrobial agents, reduces the local microbial burden and the consequent inflammatory mediator production in periodontal tissues, and its systemic anti-inflammatory effects, including reductions in circulating CRP, IL-6, fibrinogen, and HbA1c, are well-documented in randomized controlled trials [3].

In patients with concurrent endocrine disorders, these systemic anti-inflammatory benefits of periodontal treatment acquire additional clinical significance because they address the very mediators, principally IL-1β, IL-6, and TNF-α, that drive endocrine disruption through the SOCS-3-mediated insulin resistance, adiponectin suppression, HPA axis activation, and thyroid enzyme inhibition mechanisms [51]. The magnitude of these systemic benefits appears to correlate with the severity of pre-treatment periodontal inflammation and the adequacy of periodontal disease control achieved, suggesting that more thorough and more sustained periodontal disease control produces greater endocrine benefits, and that maintenance therapy designed to prevent disease recurrence is as important for systemic endocrine outcomes as it is for oral health outcomes [76, 123, 137].

Endocrine optimization represents a complementary and equally important therapeutic dimension whose periodontal benefits are supported by the molecular mechanisms reviewed [128]. In postmenopausal women, hormone replacement therapy with estrogen-containing regimens has been associated in observational studies with reduced periodontal attachment loss, improved alveolar bone density, and lower GCF inflammatory cytokine concentrations compared to untreated postmenopausal controls, consistent with the estrogen-mediated OPG induction, collagen synthesis promotion, and NF-κB suppression described in Section 3. The decision to initiate HRT should be made on the basis of the overall risk-benefit profile in each individual patient, as the systemic risks of HRT including cardiovascular events and breast cancer risk require careful consideration, but the periodontal protective effects of estrogen restoration represent a legitimate additional benefit to be weighed in this clinical calculus [194].

Vitamin D supplementation, with the goal of achieving serum 25-hydroxyvitamin D concentrations above 30 ng/mL, is a safe, inexpensive, and biologically well-justified adjunct to periodontal therapy in patients with documented deficiency, as the restoration of calcitriol-mediated antimicrobial peptide production, macrophage anti-inflammatory modulation, and OPG induction in periodontal tissues addresses multiple molecular deficits that vitamin D deficiency imposes on periodontal defense. Thyroid hormone replacement in hypothyroid patients and effective management of hyperthyroidism with antithyroid agents should be considered not only for their systemic metabolic benefits but for their potential to normalize the osteoblast-osteoclast balance and the fibroblast metabolic activity in the periodontium, as described in Section 5. In patients receiving long-term corticosteroid therapy, the prescription of periodontal maintenance programs at shorter recall intervals, the consideration of host-modulatory adjuncts including low-dose doxycycline and omega-3 fatty acids, and the evaluation of adrenal supplementation protocols for surgical periodontal procedures represent clinically important adaptations of standard periodontal care to the endocrine context.

The emerging pharmacological landscape offers additional therapeutic opportunities at the endocrine-periodontal interface that were not available even a decade ago and whose exploitation requires awareness of the molecular mechanisms linking these two domains. GLP-1 receptor agonists, whose direct anti-inflammatory and bone-protective effects in periodontal tissues were discussed in Section 4, are now prescribed at unprecedented scale for type 2 diabetes and obesity management, and their potential to reduce periodontal inflammatory indices independently of glycemic control warrants formal evaluation in dedicated randomized controlled trials.

Denosumab, the anti-RANKL monoclonal antibody approved for osteoporosis and bone metastasis management, targets the same molecular axis that is dysregulated in periodontitis-associated alveolar bone resorption, and animal model studies have shown alveolar bone preservation effects in animal models of periodontitis [169]. However, its consideration as an adjunctive periodontal agent must be accompanied by careful acknowledgment of the risk of medication-related osteonecrosis of the jaw (MRONJ), a serious and potentially debilitating complication of antiresorptive therapy that has been documented with denosumab at rates comparable to or exceeding those associated with bisphosphonates in the oncology setting. The paradoxical situation in which an agent with therapeutic potential for alveolar bone preservation carries a risk of jaw osteonecrosis underscores the need for careful patient selection, rigorous pre-treatment dental evaluation, and dedicated prospective studies in the periodontal setting before any recommendation for its adjunctive periodontal use can be made. At present, the evidence is insufficient to recommend denosumab as a periodontal adjunct; its inclusion here reflects its mechanistic relevance and the need for future clinical investigation rather than a therapeutic recommendation [195, 196].

Statins, through their pleiotropic anti-inflammatory effects including NF-κB suppression, MMP inhibition, and OPG induction in PDL cells, have shown promise as adjunctive agents in experimental and clinical periodontal studies while simultaneously benefiting the metabolic and cardiovascular comorbidities that frequently accompany endocrine disorders and periodontitis.

Resolvin E1 and other specialized pro-resolving mediators promote the active resolution of periodontal inflammation through mechanisms that include the restoration of Treg/Th17 balance [197], the enhancement of macrophage efferocytosis [198], and the reduction of RANKL expression in periodontal stromal cells [5, 170], represent a pharmacologically innovative approach to periodontal host modulation that addresses the resolution deficit characteristic of chronic periodontitis in hormonally compromised patients. The recognition that these pharmacological agents act on molecular targets that are simultaneously relevant to periodontal pathogenesis and endocrine dysfunction provides a mechanistic rationale for their evaluation as dual-purpose therapeutic interventions in patients with co-existing oral and endocrine disease.

Beyond the pharmacological agents discussed above, emerging therapeutic strategies targeting the NLRP3 inflammasome and redox-sensitive inflammatory pathways represent a frontier with particular relevance to the endocrine-periodontal interface. Small-molecule NLRP3 inhibitors, including MCC950 and its clinical derivatives, have demonstrated efficacy in preclinical models of both metabolic inflammation and experimental periodontitis, reducing IL-1β and IL-18 production, attenuating alveolar bone resorption, and improving insulin sensitivity in concurrent metabolic disease models [199, 200]. Anakinra and canakinumab, recombinant IL-1 receptor antagonist and anti-IL-1β monoclonal antibody respectively, have established clinical safety profiles in autoimmune and metabolic conditions and offer a pharmacologically validated approach to IL-1β neutralization that could be explored as an adjunct to periodontal host modulation in high-risk patients with concurrent endocrine disease. Antioxidant strategies targeting the upstream ROS-NF-κB-NLRP3 cascade, including N-acetylcysteine, mitochondria-targeted antioxidants (MitoQ, SkQ1), and pharmacological NOX inhibitors, are under active preclinical investigation for their potential to simultaneously attenuate periodontal inflammation, improve insulin signaling, and reduce endothelial oxidative stress in the context of metabolic endocrine disease. These emerging approaches, if validated in dedicated clinical trials, could complement conventional periodontal treatment and endocrine management in patients where the shared ROS-inflammasome-cytokine axis drives pathology in both disease domains.

Melatonin, a pineal hormone with broad antioxidant, anti-inflammatory, and immunomodulatory properties, represents an additional endocrine-related molecule with emerging relevance to the periodontal-endocrine interface. Melatonin receptors (MT1 and MT2) are expressed in periodontal tissues, and melatonin exerts its protective effects through several mechanisms directly relevant to this review: it scavenges reactive oxygen species and upregulates antioxidant enzymes (SOD, catalase, glutathione peroxidase), attenuating the ROS-NF-κB-NLRP3 cascade that amplifies periodontal inflammation in states of insulin resistance, hypoestrogenism, and thyroid dysfunction; it suppresses NF-κB nuclear translocation and reduces the transcription of IL-1β, IL-6, and TNF-α in periodontal macrophages and fibroblasts; and it directly inhibits NLRP3 inflammasome activation by blocking caspase-1 cleavage and IL-1β maturation. Experimentally, melatonin administration has been shown to reduce alveolar bone loss, attenuate gingival inflammatory infiltration, and improve the RANKL/OPG ratio in animal models of periodontitis; local delivery via controlled-release systems has shown promise as an adjunct to conventional periodontal therapy in preliminary clinical studies. The fact that melatonin secretion is itself disrupted by chronic inflammatory states, stress-induced HPA activation, and insulin resistance positions it as both a mechanistically relevant therapeutic candidate and a potential biomarker of circadian-endocrine dysregulation in patients with concurrent periodontal disease [201–203]

Interdisciplinary collaboration between periodontists, endocrinologists, diabetes specialists, gynecologists, and primary care physicians represents the structural prerequisite without which none of the diagnostic and therapeutic advances described above can be effectively implemented at the population level. The bidirectional molecular relationship between periodontitis and endocrine dysfunction is inherently a domain-crossing phenomenon that cannot be adequately addressed within the silos of single-specialty practice, and the clinical models that have proven most effective in analogous situations, such as the integrated diabetes-periodontics management programs that have improved glycemic outcomes in patients with both conditions, provide a template for the broader endocrine-periodontal collaboration that the evidence now supports. Shared clinical pathways, jointly developed by dental and endocrine specialty organizations, that define referral criteria, communication protocols, and shared outcome measures for patients with concurrent periodontal and endocrine disease would represent a major advance in translating the molecular science reviewed here into tangible improvements in patient care.

Medical and dental education curricula require corresponding updates to ensure that future clinicians in both fields are equipped with sufficient understanding of endocrine-periodontal interactions to recognize their clinical relevance, apply appropriate screening and referral practices, and participate meaningfully in interdisciplinary management teams. The evidence base for these educational and structural changes is now sufficiently mature to justify their implementation, and the growing burden of endocrine disorders in global populations, combined with the high prevalence of periodontitis in the same populations, makes the establishment of integrated management models an urgent public health priority as well as a clinical imperative.

## 12 Conclusions and future directions

Periodontitis and endocrine dysfunction represent two of the most prevalent chronic conditions in global populations, and the evidence synthesized in this review demonstrates that their co-occurrence is neither coincidental nor merely epidemiological. At the molecular level, these two disease domains are coupled through an extensive and highly organized network of shared signaling pathways, hub genes, and regulatory mechanisms whose architecture has been made progressively more legible through the convergence of experimental, clinical, and systems-level bioinformatics approaches. The central argument advanced throughout this review is that the endocrine-periodontal relationship is genuinely bidirectional, mechanistically grounded, and clinically actionable: hormonal dysregulation shapes periodontal susceptibility through receptor-mediated effects on immune cells, connective tissue, and alveolar bone that lower the threshold at which microbial challenge escalates to destructive inflammation, while chronic periodontal inflammation in turn disrupts endocrine homeostasis through the systemic dissemination of inflammatory mediators that impair insulin signaling, suppress adiponectin, activate the HPA axis, inhibit thyroid hormone conversion, and reduce functional vitamin D availability. These reverse effects are not trivial epiphenomena of shared risk factors; they are mechanistically specific, quantitatively significant, and partially reversible through periodontal therapy, as demonstrated by the improvements in glycemic control, adipokine profiles, and systemic inflammatory markers that follow successful periodontal disease management in patients with concurrent endocrine conditions [3, 4].

As discussed in Section 8, the RANKL/OPG axis emerges from this analysis as the molecular hub that most coherently integrates the diverse endocrine and inflammatory inputs to alveolar bone homeostasis. The central clinical implication of this convergence is that patients with multiple concurrent endocrine perturbations, such as postmenopausal women with type 2 diabetes, vitamin D deficiency, and obesity-associated adipokine dysregulation, face a compounded osteoclastogenic stimulus that exceeds what any single hormonal or inflammatory variable would predict in isolation, identifying this population as a priority group for integrated endocrine-periodontal management and for future validation of the RANKL/OPG ratio in GCF as a composite biomarker of combined hormonal and inflammatory risk.

A similar case can be made for other emerging inflammatory pathways such as NLRP3 inflammasome-driven IL-1β and IL-18 maturation whose functional and biomarker roles remain incompletely understood.

The molecular network perspective provided by the periodontitis diseasome analysis [4], detailed in Section 9, has been instrumental in revealing the systemic scope of the endocrine-periodontal relationship and in identifying the hub genes (TNF, IL6, STAT3, LEP, NOS3, VEGFA) and enriched pathways (PI3K/Akt, AGE-RAGE, JAK-STAT, Th17 differentiation, HIF-1) through which this relationship operates at the molecular level. Future research should prioritize the functional validation of these network-predicted connections through targeted experimental studies that test whether modulating specific hub gene products at the intersection of endocrine and periodontal signaling produces the predicted dual benefits in relevant disease models.

Several specific directions for future investigation emerge from the mechanistic framework developed in this review as particularly scientifically productive and clinically impactful. The periodontal effects of GLP-1 receptor agonists, now prescribed at unprecedented scale for type 2 diabetes and obesity, represent an urgent research priority given the plausible anti-inflammatory and bone-protective mechanisms of GLP-1R activation in periodontal tissues and the large patient populations already receiving these agents who also carry elevated periodontal disease risk. Dedicated randomized controlled trials evaluating GLP-1RA therapy as an adjunct to conventional periodontal treatment in patients with type 2 diabetes or obesity, with pre-specified periodontal endpoints including clinical attachment level, GCF cytokine profiles, and RANKL/OPG ratios, would provide clinically actionable evidence on whether the metabolic benefits of these agents extend meaningfully to the oral cavity. The periodontal consequences of gender-affirming hormone therapy in transgender individuals represent another area where prospective longitudinal studies are urgently needed, as the pharmacologically induced hormonal transitions experienced by these patients provide a uniquely informative natural experiment for studying the effects of sex steroids on periodontal tissues in humans, and the existing evidence base is too limited and methodologically heterogeneous to guide clinical recommendations.

Epigenetic mechanisms, including the promoter methylation of IL10 and VDR genes and the microRNA-mediated regulation of NF-κB, GR, and STAT3 signaling in periodontal cells, deserve systematic investigation as mechanistic mediators of the long-term periodontal consequences of endocrine dysregulation, and as potential therapeutic targets whose modulation through epigenetic editing or microRNA-based interventions may offer more durable benefits than conventional anti-inflammatory approaches.

The application of systems biology and precision medicine frameworks to the endocrine-periodontal relationship holds particular promise for advancing both mechanistic understanding and clinical management beyond what single-variable approaches can achieve. Multi-omics studies that simultaneously characterize the transcriptomic, proteomic, epigenomic, and metabolomic profiles of periodontal tissues in patients with specific endocrine conditions would provide an unprecedented molecular resolution of the mechanisms described in this review, potentially identifying novel molecular signatures that predict disease severity, progression risk, and therapeutic response in ways that individual biomarkers cannot.

Machine learning approaches applied to large clinical datasets integrating periodontal clinical parameters, endocrine laboratory values, adipokine profiles, inflammatory biomarkers, and genetic data have the potential to identify patient subgroups whose periodontal-endocrine molecular profiles respond differentially to specific therapeutic combinations, enabling the development of personalized management strategies tailored to individual hormonal and inflammatory contexts. The integration of oral microbiome profiling into these multi-omics frameworks is equally important, as the subgingival dysbiotic community does not merely respond passively to hormonal changes in the periodontal environment but actively participates in the disruption of endocrine homeostasis through LPS-mediated TLR signaling and molecular mimicry mechanisms whose full scope remains incompletely characterized. Longitudinal cohort studies with serial measurement of periodontal clinical parameters, subgingival microbiome composition, and endocrine biomarkers would be particularly valuable for establishing the temporal dynamics of endocrine-periodontal interactions and for identifying the windows of greatest therapeutic opportunity in the natural history of each condition.

### 12.1 Limitations

Several important limitations of the present review should be acknowledged. First, as a narrative rather than systematic review, the literature selection was structured but not exhaustive, and the possibility of selection bias toward studies consistent with the proposed mechanistic framework cannot be excluded. A formal systematic review with pre-registered PICO criteria and GRADE-level evidence assessment would provide a stronger evidentiary foundation for several of the clinical associations discussed; such a review is beyond the scope and objectives of the present work but represents an important future endeavor.

Second, the evidence supporting different endocrine-periodontal associations varies substantially in quality and quantity: while the diabetes-periodontitis relationship is supported by extensive clinical trial evidence and Mendelian randomization data, associations involving thyroid dysfunction, gender-affirming hormone therapy, and GLP-1 receptor agonist effects on the periodontium rest primarily on observational, experimental, or mechanistic evidence whose clinical translation remains to be confirmed.

Third, a substantial proportion of the mechanistic evidence reviewed derives from *in vitro* studies using supraphysiological hormone concentrations or animal models whose periodontal anatomy and immune biology differ from humans in important ways; extrapolation to human periodontal disease must therefore be made with appropriate caution.

Fourth, the majority of clinical studies reviewed are cross-sectional or of short duration, limiting causal inference and precluding characterization of the temporal dynamics of endocrine-periodontal interactions over the natural history of either condition. Fifth, the molecular network analyses informing the diseasome sections rely on gene-disease association databases that reflect the existing literature and may therefore overrepresent well-studied conditions and underrepresent associations for which investigation has been limited.

Finally, the potential for publication bias in the periodontal literature—particularly in intervention studies—should be considered when interpreting the consistency of positive findings reviewed herein.

### 12.2 Conclusions

The translation of the mechanistic insights synthesized in this review into clinical practice ultimately depends on structural changes in healthcare delivery, professional education, and research funding priorities that recognize the bidirectional endocrine-periodontal relationship as a genuine interdisciplinary challenge. Clinical practice guidelines for the management of diabetes, thyroid disease, postmenopausal osteoporosis, PCOS, and adrenal disorders should formally incorporate recommendations for periodontal screening and referral, acknowledging the evidence that uncontrolled periodontitis is an active modifier of endocrine disease management outcomes. Periodontal clinical guidelines should correspondingly include endocrine status as a formal component of periodontal risk assessment, specifying the endocrine conditions and therapeutic exposures that most significantly modify periodontal disease susceptibility and treatment response. Research funding agencies should prioritize interdisciplinary grant mechanisms that bring together periodontists, endocrinologists, immunologists, and computational biologists to address the endocrine-periodontal interface with the methodological rigor and mechanistic depth that the complexity of the relationship demands. Medical and dental education curricula require updates that ensure graduates of both professions possess sufficient understanding of endocrine-periodontal interactions to recognize their clinical relevance in patient care, initiate appropriate screening and referral, and communicate effectively across disciplinary boundaries. These structural changes are not aspirational ideals but practical necessities given the high and growing prevalence of both endocrine disorders and periodontitis in aging global populations and the compelling evidence that their bidirectional molecular coupling produces clinical outcomes in each domain that cannot be optimally managed without attention to the other.

In conclusion, the relationship between periodontal disease and endocrine imbalance is grounded in a rich and increasingly well-characterized molecular landscape whose full clinical significance is only now beginning to be appreciated. The convergence of estrogen, progesterone, androgen, insulin, adipokine, thyroid hormone, glucocorticoid, and mineral-regulating hormone signals on the shared inflammatory and bone remodeling machinery of the periodontium, and the reverse disruption of endocrine homeostasis by the systemic inflammatory consequences of periodontal infection, together constitute a bidirectional molecular relationship of genuine clinical importance.

A similar case can be made for the NLRP3 inflammasome, which emerges from this analysis as a central integrator of metabolic danger signals, microbial pattern recognition, and endocrine inputs simultaneously dysregulated in periodontitis and endocrine disorders. NLRP3-driven IL-1β and IL-18 maturation connects insulin resistance (via AGE-RAGE and ROS), vitamin D deficiency (via loss of VDR-mediated transcriptional suppression), glucocorticoid dysregulation, and thyroid hormone imbalance to the amplification of periodontal inflammatory responses, and represents both a biomarker target and a therapeutic opportunity whose full clinical relevance remains to be established in dedicated trials.

The systems-level analysis of the periodontitis diseasome has provided a molecular map of this relationship that confirms its biological depth and identifies specific hub genes and pathways as targets for integrated therapeutic strategies. Moving from this mechanistic understanding to improved patient outcomes will require interdisciplinary collaboration, precision diagnostic tools, innovative clinical trial designs, and structural changes in healthcare delivery that transcend the boundaries of single specialties. The molecular science is sufficiently advanced to justify these investments, and the patient populations who stand to benefit from integrated endocrine-periodontal management are large, growing, and currently underserved by the discipline-siloed models of care that predominate in clinical practice. Advancing toward more integrated, molecularly informed, and patient-centered approaches to the co-management of periodontal and endocrine disease represents both a scientific imperative and a meaningful opportunity to reduce the burden of chronic disease in vulnerable populations worldwide.

## Glossary

List of abbreviations used in Figures 1, 2, and 3.

**Table:**

Abbreviation	Full term
ACTH	Adrenocorticotropic hormone
AGE	Advanced glycation end-product
AdipoR	Adiponectin receptor (R1/R2)
Akt	Protein kinase B
AP-1	Activator protein 1
AR	Androgen receptor
anti-TPO	Anti-thyroid peroxidase antibody
BBB	Blood-brain barrier
cAMP	Cyclic adenosine monophosphate
CRH	Corticotropin-releasing hormone
DIO1	Type 1 deiodinase
ERα/ERβ	Estrogen receptor alpha/beta
FOXO1	Forkhead box protein O1
GLP-1	Glucagon-like peptide-1
GR	Glucocorticoid receptor
HIF-1	Hypoxia-inducible factor 1
HPA	Hypothalamic-pituitary-adrenal axis
IL-1β	Interleukin-1 beta
IL-6	Interleukin-6
IL-10	Interleukin-10
IL-17	Interleukin-17
IR	Insulin receptor
IRS-1	Insulin receptor substrate 1
1α-OHase	25-hydroxyvitamin D${}_{3}$ 1α-hydroxylase
JAK2	Janus kinase 2
LEP-R	Leptin receptor
LL-37	Cathelicidin antimicrobial peptide
LPS	Lipopolysaccharide
MMP-1	Matrix metalloproteinase-1
MMP-3	Matrix metalloproteinase-3
MMP-8	Matrix metalloproteinase-8
NFATc1	Nuclear factor of activated T cells 1
NF-κB	Nuclear factor kappa-light-chain-enhancer of activated B cells
OBL	Osteoblast
OCL	Osteoclast
OPG	Osteoprotegerin
PDL	Periodontal ligament
PGE2	Prostaglandin E2
PI3K	Phosphoinositide 3-kinase
PKA	Protein kinase A
PPARα	Peroxisome proliferator-activated receptor alpha
PTH	Parathyroid hormone
PTH1R	Parathyroid hormone receptor 1
RAGE	Receptor for advanced glycation end-products
RANKL	Receptor activator of NF-κB ligand
RUNX2	Runt-related transcription factor 2
SERM	Selective estrogen receptor modulator
SOCS-3	Suppressor of cytokine signaling 3
STAT3	Signal transducer and activator of transcription 3
T3	Triiodothyronine
T4	Thyroxine
TLR4	Toll-like receptor 4
TNF-α	Tumor necrosis factor alpha
TNFR1	Tumor necrosis factor receptor 1
TPO	Thyroid peroxidase
TRα/TRβ	Thyroid hormone receptor alpha/beta
VDR	Vitamin D receptor
VEGFA	Vascular endothelial growth factor A
Wnt/β-cat	Wnt/beta-catenin signaling pathway
